# Genetic relationships between sympatric and allopatric *Coregonus* ciscoes in North and Central Europe

**DOI:** 10.1186/s12862-021-01920-8

**Published:** 2021-10-06

**Authors:** Thomas Mehner, Stefan Palm, Bo Delling, Juha Karjalainen, Jolanta Kiełpińska, Asja Vogt, Jörg Freyhof

**Affiliations:** 1 Leibniz Institute of Freshwater Ecology and Inland Fisheries, Müggelseedamm 310, 12587 Berlin, Germany; 2 Department of Aquatic Resources, Institute of Freshwater Research, Swedish University of Agricultural Sciences, Drottningholm, Sweden; 3 Department of Zoology, Swedish Museum of Natural History, Stockholm, Sweden; 4 Department of Biological and Environmental Science, University of Jyväskylä, Jyväskylä, Finland; 5 Department of Aquatic Bioengineering and Aquaculture, Faculty of Food Science and Fisheries, West Pomeranian University of Technology in Szczecin, Szczecin, Poland; 6 Museum Für Naturkunde, Leibniz Institute for Evolution and Biodiversity Science, Berlin, Germany

**Keywords:** *Coregonus albula*, Post-glacial divergence, Baltic cisco complex, Microsatellites, Species loss

## Abstract

**Background:**

Sympatric speciation along ecological gradients has been studied repeatedly, in particular in freshwater fishes. Rapid post-glacial ecological divergence has resulted in numerous endemic species or ecologically distinct populations in lakes of the temperate zones. Here, we focus on the Baltic cisco (*Coregonus albula*) complex, to study the genetic similarity among two pairs of sympatric autumn- and spring-spawning populations from post-glacial German Lakes Stechlin and Breiter Luzin. For comparison, we included a similar pair of sympatric populations from the Swedish Lake Fegen. We wanted to explore potential genetic similarities between the three sympatric cisco population pairs in the three lakes, to evaluate whether the pairs may have emerged independently in the three lakes, or whether two different species may have colonized all three lakes independently. Furthermore, we considered allopatric *C. albula* populations from three Polish, three Finnish, and four Swedish locations, added one Siberian population of the sister species *C. sardinella* and a Swedish *C. maraena* (whitefish) population. By genotyping nine microsatellite markers in 655 individuals from these 18 populations, we wanted to elucidate how strongly the cisco populations differ across a larger geographical area within Europe. Finally, we compared the genetic differences between the spring- and autumn-spawning populations of ciscoes in the two German lakes to infer the potentially deteriorating effect of strong anthropogenic pressure on the lakes.

**Results:**

Dendrogram, Principal Coordinate Analysis and admixture analysis all indicated strong correspondence between population differentiation and geographical location for most cisco populations in Europe, including the Siberian population of *C. sardinella*. However, populations from some Swedish lakes deviated from this general pattern, by showing a distinct genetic structure. We found evidence for independent evolution of the three sympatric population pairs, because the populations co-occurring in the same lake were always most closely related. However, genetic differentiation was weak in the two German population pairs, but strong in the Swedish Lake Fegen, indicating that the weak differentiation in the German pairs reported earlier has eroded further.

**Conclusions:**

Our results suggest that the genetic differentiation at neutral genetic markers among populations of the Baltic cisco complex has evolved (and is maintained) by random genetic drift in isolated populations. However, earlier studies on the Swedish populations combining mitochondrial DNA and microsatellite data indicate that also post-glacial immigration from separate glacial refugia has shaped the present genetic population structure. The low neutral differentiation of the German sympatric pairs in contrast to the Swedish pair suggests that recent anthropogenic effects on the lakes in Germany may put the endemic spring-spawners at risk to extinction.

**Supplementary Information:**

The online version contains supplementary material available at 10.1186/s12862-021-01920-8.

## Background

Traditionally, the process of speciation has been classified by using a geographical context. The allopatric mode is characterized by extrinsic barriers during divergence, the parapatric mode includes a partial extrinsic barrier, while the sympatric mode does not involve any extrinsic barriers [1]. However, more recent discussions consider speciation as a process along axes of spatial, reproductive and ecological differentiation [2]. Accordingly, allopatric and sympatric speciation modes are the extreme ends of a continuum, whereas most of the examples for speciation processes likely include allopatric, parapatric and sympatric phases [1]. One of the speciation modes that have attracted frequent research interests is sympatric speciation along ecological gradients [3–5]. Several examples of speciation in ecological contexts exist for insects [6], reptiles [7], birds [8] and fishes [9].

Freshwater fishes are good model systems for post-glacial ecological divergence in sympatry [3, 10, 11]. Famous examples are the morphological and genetic divergence between benthic and limnetic sticklebacks (*Gasterosteus*), Arctic charrs (*Salvelinus*) and smelts (*Osmerus*) [12–14]. The Holarctic genus *Coregonus* (whitefishes and ciscoes) is particularly diverse [15], with numerous examples of sympatric pairs or species flocks that have evolved after the last glaciations [16–18]. *Coregonus* are considered typical examples for incipient and ongoing speciation events in post-glacial lakes, induced by segregation along ecological gradients, such as lake habitats (littoral, pelagic and profundal habitats) and feeding niches (planktivory, benthivory, and occasionally piscivory), by physiological differences along vertical temperature gradients, or by predator-induced morphological divergence [16, 19, 20]. It is generally assumed that similar trajectories of ecological divergence found in different lakes would support independent sympatric speciation from a single ancestor [21]. However, alternative speciation modes are also discussed, involving sequential invasion and (often) hybridisation of two species or lineages, reinforcing sympatric speciation [16]. According to the young age of the species and the often low morphological and genetic divergence, there are also several documented cases of reversed speciation in *Coregonus*, when sympatric species went extinct due to the deterioration of ecological gradients, for example following lake eutrophication, species invasion or fisheries [22, 23]. Indeed, the great diversity in *Coregonus* is at risk with respect to the conservation of evolutionarily significant units in several lakes, and many extinction cases have been documented [15, 23]. Similar speciation reversals have been observed in cichlids (genus *Haplochromis*) [24], sticklebacks (*Gasterosteus*) [25] and shads (*Alosa*) [26].

This risk seems also substantial for some populations of the Baltic ciscoes (*C. albula* complex). Baltic ciscoes have smaller maximum size and age than whitefish and live predominantly in post-glacial lakes in Scandinavia, Finland, Poland and North Germany. Cisco species flocks are not known, but several sympatric population pairs have been described from lakes in Germany and Sweden [27–29], characterized by the coexistence of the autumn-spawning vendace *C. albula* and a second form, which spawns in spring. The spring-spawning cisco in German Lake Breiter Luzin has been described as *C. lucinensis* [27], whereas the spring-spawners from German Lake Stechlin have been described as *C. fontanae* [29]. In Sweden, the spring-spawners from Lakes Fegen, Ören, Stora Hålsjön and Åsunden were described as *C. trybomi* [28]. Sympatric cisco pairs in Finnish lakes were likewise characterized by the coexistence of an autumn- and a winter- or spring-spawning population [30, 31], and sympatric populations seem to be still abundant at least in Lakes Sokojärvi and Kajoonjärvi in Eastern Finland. A different type of sympatric pairs is found in Russia where large piscivorous ciscoes coexist with *C. albula* in the large Lakes Ladoga (*C. ladogae*) and Onega (*C. kilez*) [27].

Recently, the population differentiation based on microsatellite markers and mitochondrial DNA of several Swedish *C. albula* populations, including the only remaining sympatric populations from Lake Fegen, has comprehensively been studied [32, 33]. Two distinct clades (mtDNA) could be differentiated with their sets of haplotypes covarying with two distinct population assemblages (microsatellites). One of the assemblages detected exist in southern higher-elevation lakes (assemblage I) and in lower-elevation Swedish lakes (assemblage II), respectively. Assemblage I was likely formed when the ice retreated from south to north (~ 12.000 years ago), and fish from the southern refuge area could populate the Baltic basin including the southern Swedish lakes [32]. The second assemblage in Sweden may originate from another glacial refugium, connected to the Baltic basin about 1000 years later than when the first immigration wave occurred from the south. The mtDNA clade II was further present in Finland and Germany [32, 34]. Both clades were present in ciscoes of Lake Breiter Luzin [34]. Furthermore, these studies demonstrate that microsatellite alleles of Baltic *C. albula* and circumpolar *C. sardinella* are present  in the two population assemblages and hence both species do not form two fully independent groups, but have experienced introgressive hybridization in their evolutionary past.

Previous analyses of genetic relationships between the German sympatric ciscoes have resulted in partly different evolutionary scenarios. By using six microsatellites and part of the mitochondrial DNA, Schulz et al. [34] found higher similarity between the sympatric pairs than between either spring- or autumn-spawners from the different lakes, suggesting independent origin of both pairs. In contrast, by using > 1200 Amplified Fragment Length Polymorphism (AFLP) markers, genetic similarity was higher between spring-spawners and autumn-spawners across the lakes, than for the sympatric populations within the lakes [35]. However, when loci under putative selection were excluded, the genetic relationships between the four populations resembled more the model of independent evolution in both lakes as suggested by Schulz et al. [34].

For the conservation of evolutionarily significant units, it is decisive to discriminate between a scenario that all spring-spawning populations have evolved independently and hence are endemic for each of the lakes, and a scenario by which two different ancestral genetic lineages have invaded all lakes with sympatric populations, suggesting that all spring-spawners would have a common evolutionary origin. However, three out of originally four Swedish sympatric population pairs have become extinct during the last decades [32, 33], presumably by anthropogenically induced effects on their lakes. In Sweden, autumn- and spring-spawners of the *C. albula* complex likely coexist not anywhere else than in the Lake Fegen. The situation in Finland remains to be studied further. In Germany, sympatric pairs seem to have evolved only in the two Lakes Stechlin and Breiter Luzin. The genetic differentiation between the autumn- and spring-spawners in both lakes was weak according to earlier studies [34]. Furthermore, both lakes underwent strong eutrophication during the last decades [36–38]. Therefore, the putatively endemic spring-spawners in both lakes may be exposed to a high risk of extinction.

We used the microsatellite marker set developed for Swedish *C. albula* [32] as a framework to allow direct comparisons of neutral nuclear genetic data between the Swedish cisco populations and newly created data from allopatric and sympatric cisco populations around the Baltic Sea, to infer the potential correspondence between geographical location and genetic differentiation. In particular, we wanted to compare the phylogeography of the remaining sympatric populations of Baltic ciscoes from Sweden with those from the two German lakes, in relation to several allopatric *C. albula* populations from Poland, Finland and Sweden. Our study had three aims. First, we wanted to elucidate how strongly the cisco populations differ across a larger geographical area including populations from Germany, Poland, Sweden and Finland, and by including a population of the geographically most distant Siberian *C. sardinella*. Second, we wanted to explore potential genetic similarities between the three sympatric cisco population pairs in the three lakes, to evaluate whether the pairs may have emerged independently in the three lakes, or whether two different species may have colonized the three lakes. Third, we wanted to compare the differences in genetic structure between the spring- and autumn-spawning populations of ciscoes within both German Lakes Stechlin and Breiter Luzin with similar data obtained about 20 years ago, to infer the potentially deteriorating effect of a strong anthropogenic pressure on these lakes.

## Results

### Genetic structure within populations

**Fig. 1:**
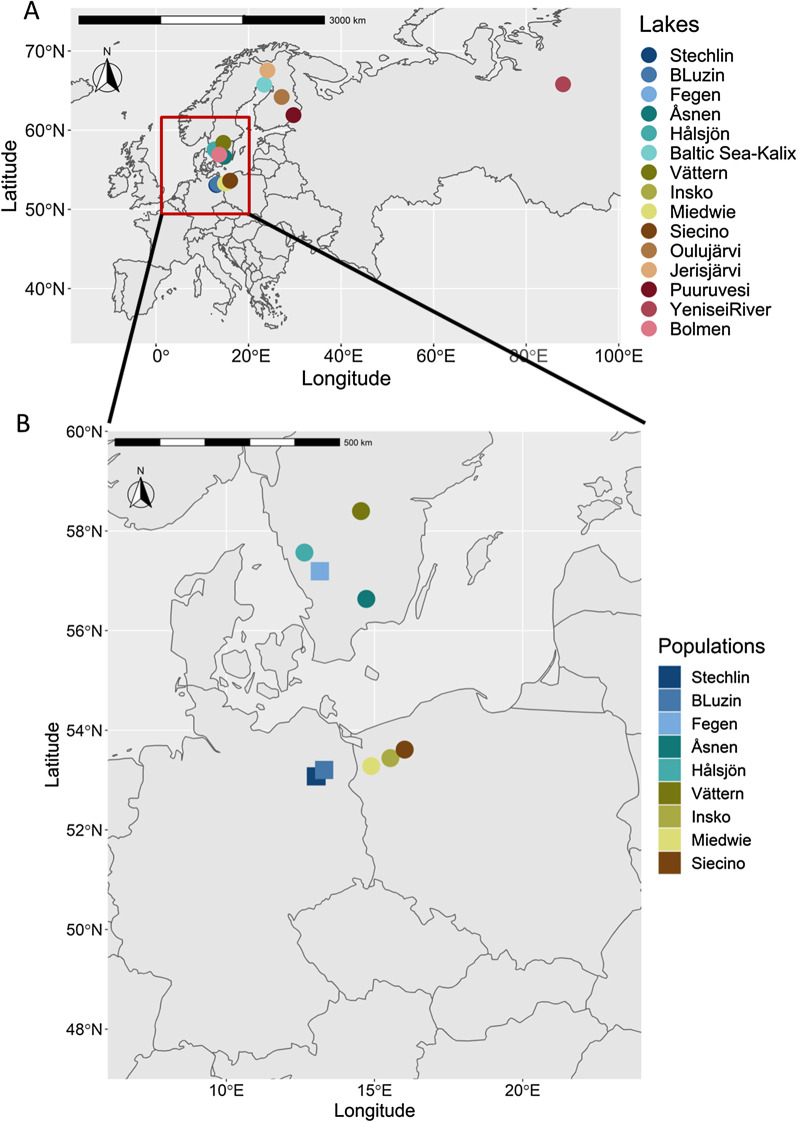
Geographical map of sampling locations. Map of the 15 locations of *Coregonus* sampled in Europe (**A**) and detailed map of lakes sampled around the southern Baltic Sea (Sweden, Germany and Poland), with squares = sympatric populations, and dots = allopatric populations (**B**)

In total, we found 5.1% of missing genotypes among the nine microsatellites in the 18 *Coregonus* population samples (Additional file 1: Table S1). The highest proportion of missing data was found for the markers *Sfo8* (11.7%) and *BWF1* (7.8%) and for the Finnish *C. albula* populations from Lakes Oulujärvi (19%) and Jerisjärvi (29%) (Additional file 1: Table S1). Among the 703 individual fishes sampled from the 18 populations, 48 fishes (6.8%) had more than two loci missing (exclusively found in the populations from Germany, Poland, Finland and Russia). Accordingly, we included only 655 individuals (range N = 10 to 79 per population) with genotypes from at least seven microsatellite loci into the subsequent statistical analyses (Table 1, Fig. 1), whereof 563 (86%) had a complete set of genotypes at all loci.

**Table 1:** Overview on *Coregonus* populations sampled in five countries

Population origin	Country	Species	N	A_R_	F_IS_	P Het
Lake Stechlin	Germany	*C. albula*	65 (67)	5.5	0.092	< 0.0001
Lake Stechlin	Germany	*C. fontanae*	57 (67)	5.4	0.163	< 0.0001
Lake Breiter Luzin	Germany	*C. albula*	25 (29)	5.2	0.082	0.0351
Lake Breiter Luzin	Germany	*C. lucinensis*	27 (30)	5.3	0.040	0.004
Lake Fegen	Sweden	*C. albula* AS	70 (70)	4.0	− 0.011	0.5799
Lake Fegen	Sweden	*C. albula* SS	79 (79)	3.4	0.023	0.1844
Lake Stora Hålsjön	Sweden	*C. albula*	50 (50)	4.6	− 0.005	0.8836
Lake Åsnen	Sweden	*C. albula*	40 (40)	3.5	− 0.008	0.7656
Baltic Sea (Kalix)	Sweden	*C. albula*	32 (32)	5.5	0.045	0.0569
Lake Vättern	Sweden	*C. albula*	32 (32)	5.4	0.018	0.0955
Lake Ińsko	Poland	*C. albula*	30 (30)	5.1	0.217	< 0.0001
Lake Miedwie	Poland	*C. albula*	31 (31)	5.7	0.102	0.0001
Lake Siecino	Poland	*C. albula*	30 (30)	5.4	0.028	0.0809
Lake Oulujärvi	Finland	*C. albula*	22 (32)	5.5	0.116	0.0011
Lake Jerisjärvi	Finland	*C. albula*	10 (19)	4.4	0.185	0.0013
Lake Puruvesi	Finland	*C. albula*	24 (32)	5.9	0.237	< 0.0001
River Yenisei	Russia	*C. sardinella*	13 (15)	6.3	− 0.003	0.5019
Lake Bolmen	Sweden	*C. maraena*	18 (18)	4.7	0.073	0.1363
Total			655 (703)			

Lake or river origin, scientific name, sample size as number of individuals with no more than two loci missing (in brackets: number of individuals analysed in total) (N), mean allelic richness normalized to a sample size of N = 10 (A_*R*_), inbreeding coefficient (*F*_*IS*_), P-values for heterozygote deficits (P Het)

*AS* autumn-spawner, *SS* spring-spawner

Genetic structure of samples of the sympatric populations from German lakes obtained in different years did not differ significantly for most combinations (Additional file 1: Tables S2, S3). Therefore, the samples from these populations were pooled across years. However, weak differentiation was found between *C. albula* samples taken in Lake Stechlin in 2011 vs. those taken in 2018, indicating some temporal genetic change.

All microsatellites were variable in all populations, with nine (*Cisco126*, *Str73*) to 72 (*BWF2*) alleles per locus across all populations (Additional file 1: Fig. S1). Allelic richness A_R_, the mean number of alleles per locus and population adjusted to the minimum N = 10, ranged from 3.4 to 6.3 (Table 1). The lowest mean value (3.4) was found in Lake Fegen spring-spawners, whereas the highest value (6.3) existed in *C. sardinella* from Yenisei River. In line with Delling et al. [32], A_R_ was lower in the Swedish assemblage I (Fegen autumn- and spring-spawners, Åsunden, Stora Hålsjön; 3.4–4.6) compared to assemblage II (Kalix, Vättern; 5.4–5.5). A_R_ in all Finnish, German and Polish cisco samples was similar to the level in Swedish assemblage II (5.1–5.9), except for Lake Jerisjärvi (4.4), where A_R_ was similar as in Swedish assemblage I (Table 1).

In nine of the 18 populations, the expected heterozygosity across all loci was significantly higher than the observed heterozygosity (i.e. F_is_ > 0, see Table 1). The number of deviations from HWE tested individually for all nine analyzed microsatellite loci and corrected by the false discovery rate for multiple tests varied between populations (Additional file 1: Table S4). *C. albula* from Lakes Stechlin and Ińsko had five out of nine loci each deviating from HWE, whereas *C. albula* from Lakes Fegen (both autumn- and spring-spawners), Stora Hålsjön, Oulujärvi and Jerisjärvi, *C. maraena* from Lake Bolmen and *C. sardinella* from Yenisei River did not show any deviation from HWE (Additional file 1: Table S4). Most deviations from HWE occurred in the markers *BWF1* (N = 7 populations) and *Cocl23* (N = 5).

### Genetic structure between populations

Pairwise θ was significantly larger than zero for all population pairs except between the sympatric populations from Lake Breiter Luzin (θ = − 0.0012) (Additional file 1: Table S5). However, similarly weak differentiation was found between the sympatric populations in Lake Stechlin (θ = 0.005), between Lake Vättern and the Baltic population near Kalix (θ = 0.013), and between the Polish Lakes Miedwie and Siecino (θ = 0.012). The AMOVA splitting the total genetic variation among the 18 contemporary populations indicated that the among-populations variance (13.7%) was substantially higher than the among-individuals within-populations variance (6.1%), whereas the variation within individuals represented the greatest share (80.2%) (Table 2).

**Table 2:** Analysis of molecular variance for 655 *Coregonus* individuals originating from either 18 contemporary populations sampled for this study (upper subtable) or assigned to 6 putative ancestral groups (lower subtable), as defined by LEA

	Df	Sum sq	Mean sq	Sigma	Percent	P
18 contemporary populations						
Between pops	17	1069.1	62.9	0.8	13.7	0.01
Between samples within pops	637	3457.1	5.4	0.4	6.1	0.01
Within samples	655	3087.5	4.7	4.7	80.3	0.01
Total	1309	7613.7	5.8	5.9	100.0	
6 putative ancient groups						
Between pops	5	700.8	140.2	0.7	12.1	0.01
Between samples within pops	649	3825.5	5.9	0.6	9.8	0.01
Within samples	655	3087.5	4.7	4.7	78.1	0.01
Total	1309	7613.7	5.8	6.0	100.0	

**Fig. 2:**
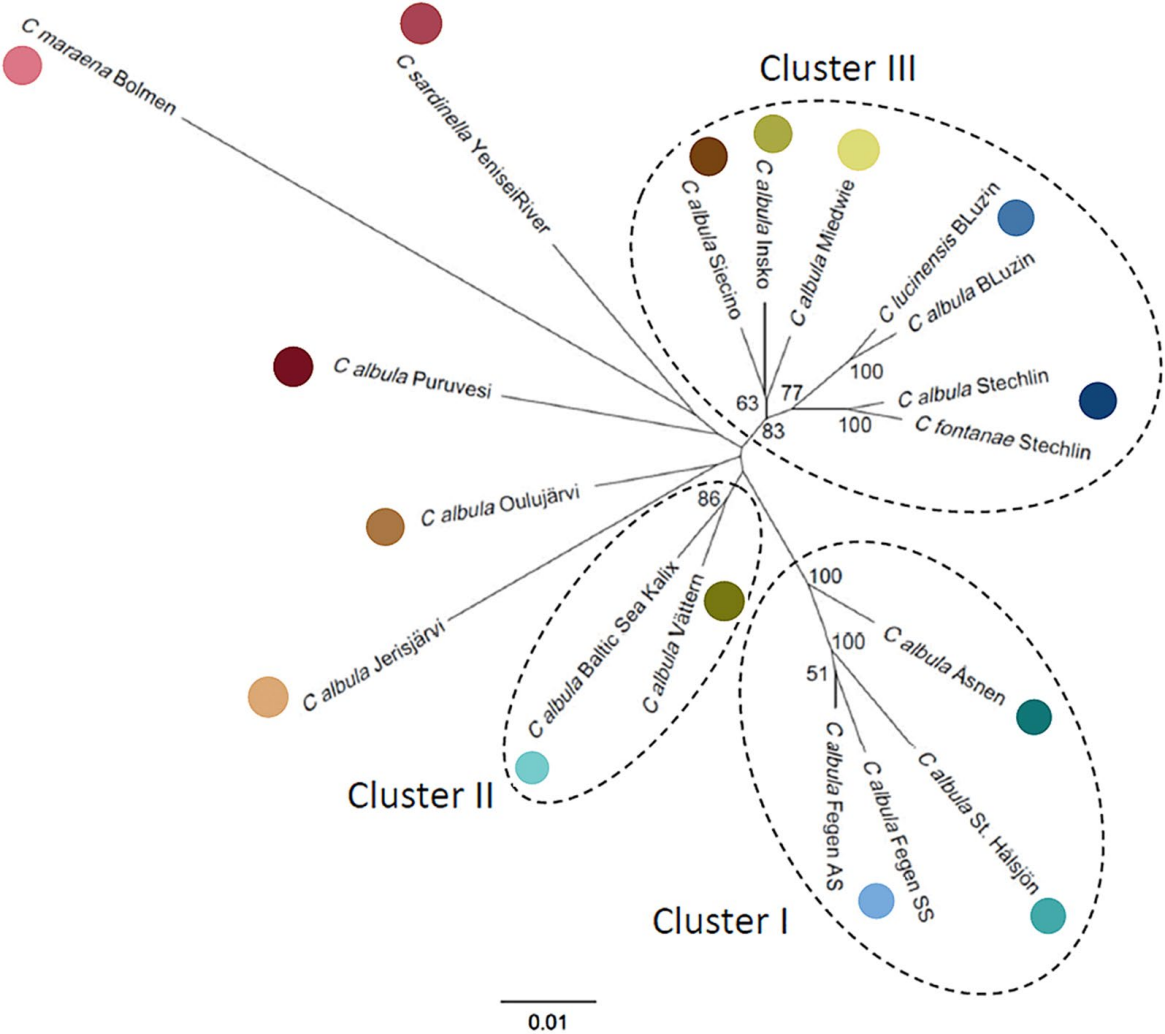
Dendrogram of sampled populations. Neighbor-joining dendrogram (nine microsatellites) including 18 sympatric and allopatric *Coregonus* populations from 15 sampling locations in Germany, Sweden, Poland, Finland and Russia (in total 655 individuals; cf. Table 1). *AS* autumn spawner, *SS* spring spawner. Only bootstrap values > 50% are shown. Three main clusters of populations, as discussed in the text, are displayed (Cluster I and II are corresponding to the population assemblages I and II identified by Delling et al. [20]). The colored dots refer to the sampling locations in Fig. 1

Similarities of the 18 *Coregonus* populations were depicted as a neighbor-joining dendrogram based on the Chord distance (Fig. 2). The sympatric populations in Lakes Stechlin (bootstrap value 100%), Breiter Luzin (100%) and Fegen (51%) each grouped together. The dendrogram was composed of three main population clusters, based on bootstrap support > 80% for the main nodes: (1) three Swedish lakes including the sympatric populations of Lake Fegen and the allopatric populations from Lakes Stora Hålsjön and Åsnen (belonging to the Swedish microsatellite assemblage I; [32]), (2) the allopatric *C. albula* populations in the Swedish Lake Vättern and the Baltic Sea population near Kalix (belonging to the microsatellite assemblage II; [32]), and (3) the three allopatric *C. albula* populations from Lakes Ińsko, Miedwie and Siecino in western Poland combined with the two sympatric population pairs in German Lakes Stechlin and Breiter Luzin (Fig. 2). The allopatric *C. albula* from Finnish Lakes Oulujärvi and Jerisjärvi were grouped together, whereas *C. albula* in Lake Puruvesi tended to group with *C. sardinella* from Yenisei River and *C. maraena* in Lake Bolmen, but with weakly supported bootstrap values (< 50%) (Fig. 2).

**Fig. 3:**
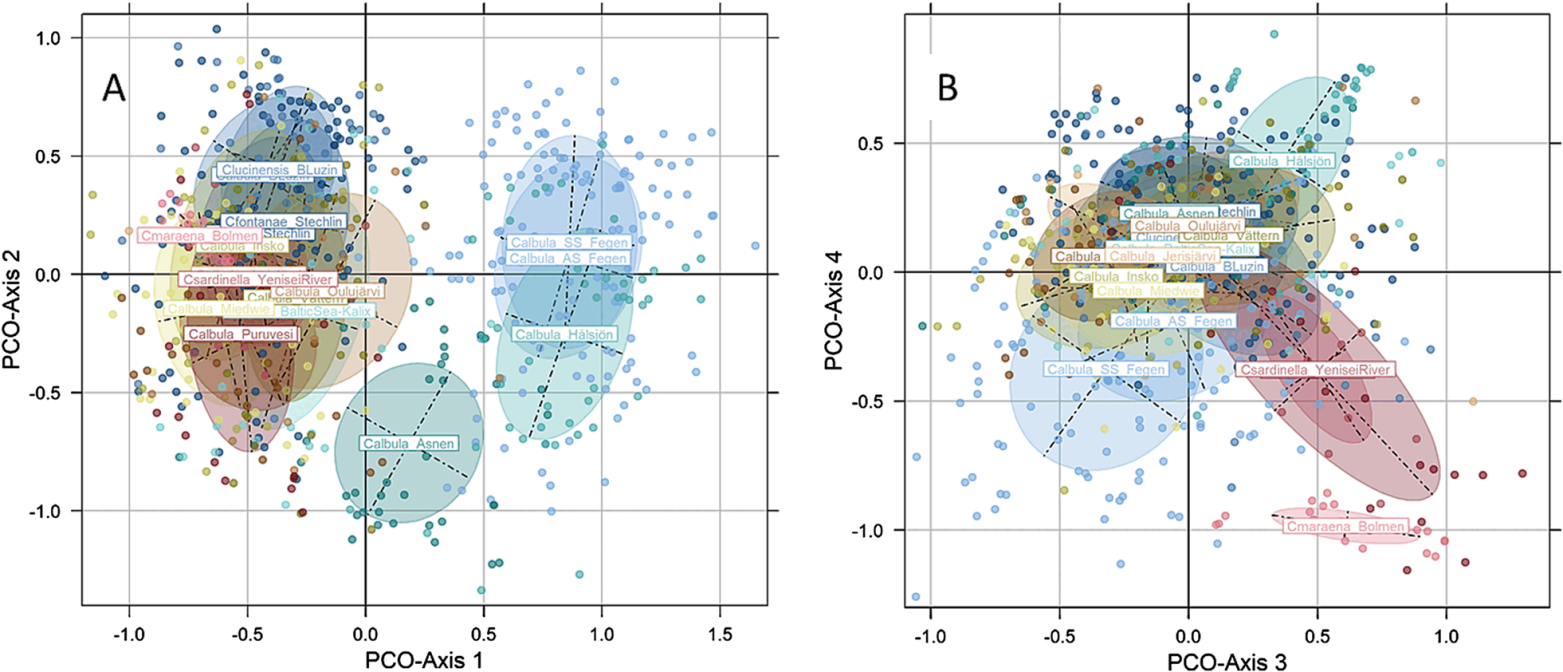
Biplots of Principal Coordinate Analysis. Biplots of the dimension reduction by principal component analysis (PCoA) of 655 *Coregonus* individuals (dots) and inertia ellipses from 18 populations, with **A** PCoA-axes 1 and 2, and **B** PCoA-axes 3 and 4 displayed. The colors of dots and population names follow the coloring in Figs. 1 and 2

The dimension reduction by PCoA of the genetic data of 655 individuals predicted a total of 28.5% variance along the first four axes (11.7%, 6.6%, 5.3%, 4.9%, respectively). In the reduced space along PCoA-Axes 1 and 2, the major split between the populations of cluster I versus clusters II and III was confirmed, similar to the dendrogram (Fig. 3A; see Fig. 2 for comparison). For better resolving the location of individual populations in the reduced space, their distributions along axes 1 and 2 are displayed separately (Additional file 1: Fig. S2). Along PCoA-axes 3 and 4, the populations of *C. albula* from Lake Puruvesi, *C. sardinella* and *C. maraena* separated from the Baltic cisco populations, similar to the split of populations in the dendrogram (Fig. 3B).

**Fig. 4:**
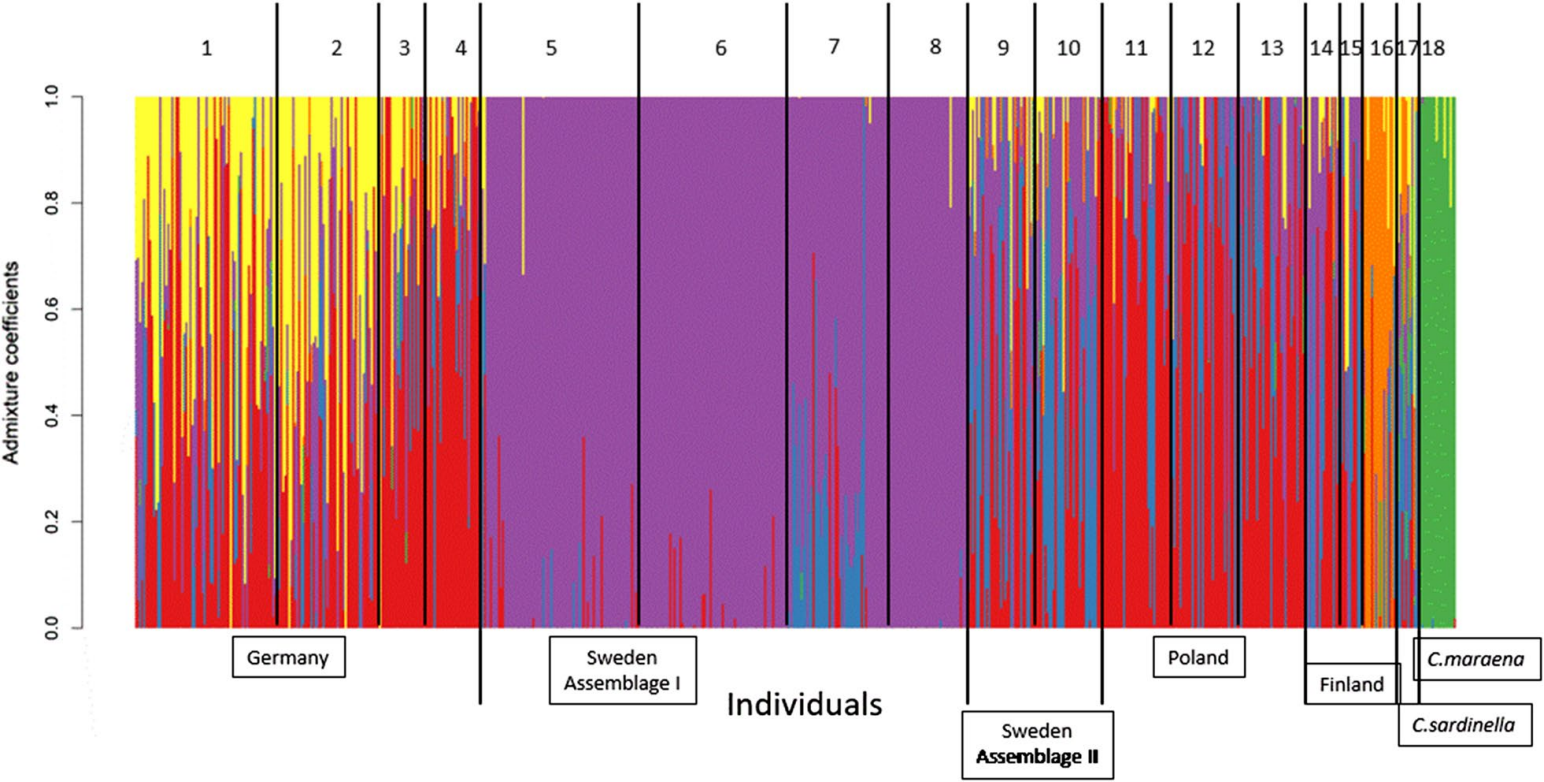
Membership probabilities of sampled individuals. Individual membership probabilities to six putative ancestral genetic groups of 655 individuals from the 18 sympatric and allopatric populations in Germany, Sweden, Poland, Finland and Russia, as obtained by the R-package LEA. Columns represent individuals and the different colors within each column represent the probability of membership of that individual to one of the six groups suggested by the cross-entropy criterion *K*. The numbers above the groups of columns represent the populations: 1 = Lake Stechlin *C. albula*, 2 = Lake Stechlin *C. fontanae*, 3 = Lake Breiter Luzin *C. albula*, 4 = Lake Breiter Luzin *C. lucinensis*, 5 = Lake Fegen *C. albula* autumn-spawner, 6 = Lake Fegen *C. albula* spring-spawner, 7 = *C. albula* Lake Stora Hålsjön, 8 = *C. albula* Lake Åsnen, 9 = *C. albula* Baltic Sea near Kalix, 10 = *C. albula* Lake Vättern, 11 = *C. albula* Lake Ińsko, 12 = *C. albula* Lake Miedwie, 13 = *C. albula* Lake Siecino, 14 = *C. albula* Lake Oulujärvi, 15 = *C. albula* Lake Jerisjärvi, 16 = *C. albula* Lake Puruvesi, 17 = *C. sardinella* Yenisei River, 18 = *C. maraena* Lake Bolmen

Following admixture analyses (LEA), the cross-entropy criterion *K* was lowest for *N* = 6 ancestral genetic groups (Additional file 1: Fig. S3). The admixture coefficients for these six groups were strongly scattered across the 18 populations (Fig. 4). The whitefish *C. maraena* population from Lake Bolmen was clearly distinct and was composed of individuals with admixture coefficients well distinct from the Baltic cisco populations. Low levels of admixture were likewise found for the populations from the Swedish microsatellite assemblage I (Lakes Fegen, Stora Hålsjön and Åsnen) and the Finnish Lake Puruvesi. In contrast, the German sympatric populations from Lakes Stechlin and Breiter Luzin, the three Polish populations and the two Finnish populations from Lakes Jerisjärvi and Oulujärvi showed high levels of admixture (Fig. 4).

The AMOVA, splitting the variance among the six ancestral groups identified above, indicated that the among-populations variance (12.1%) was higher than the among-individuals within-populations variance (9.8%), whereas the variation within individuals represented the greatest share (78.1%) (Table 2). However, the variance decomposition was very similar to that obtained for the 18 contemporary populations, with even slightly higher heterogeneity found among individuals within the populations for the 6 ancestry groups than for the 18 contemporary populations (9.8% and 6.1%, respectively, Table 2).

**Fig. 5:**
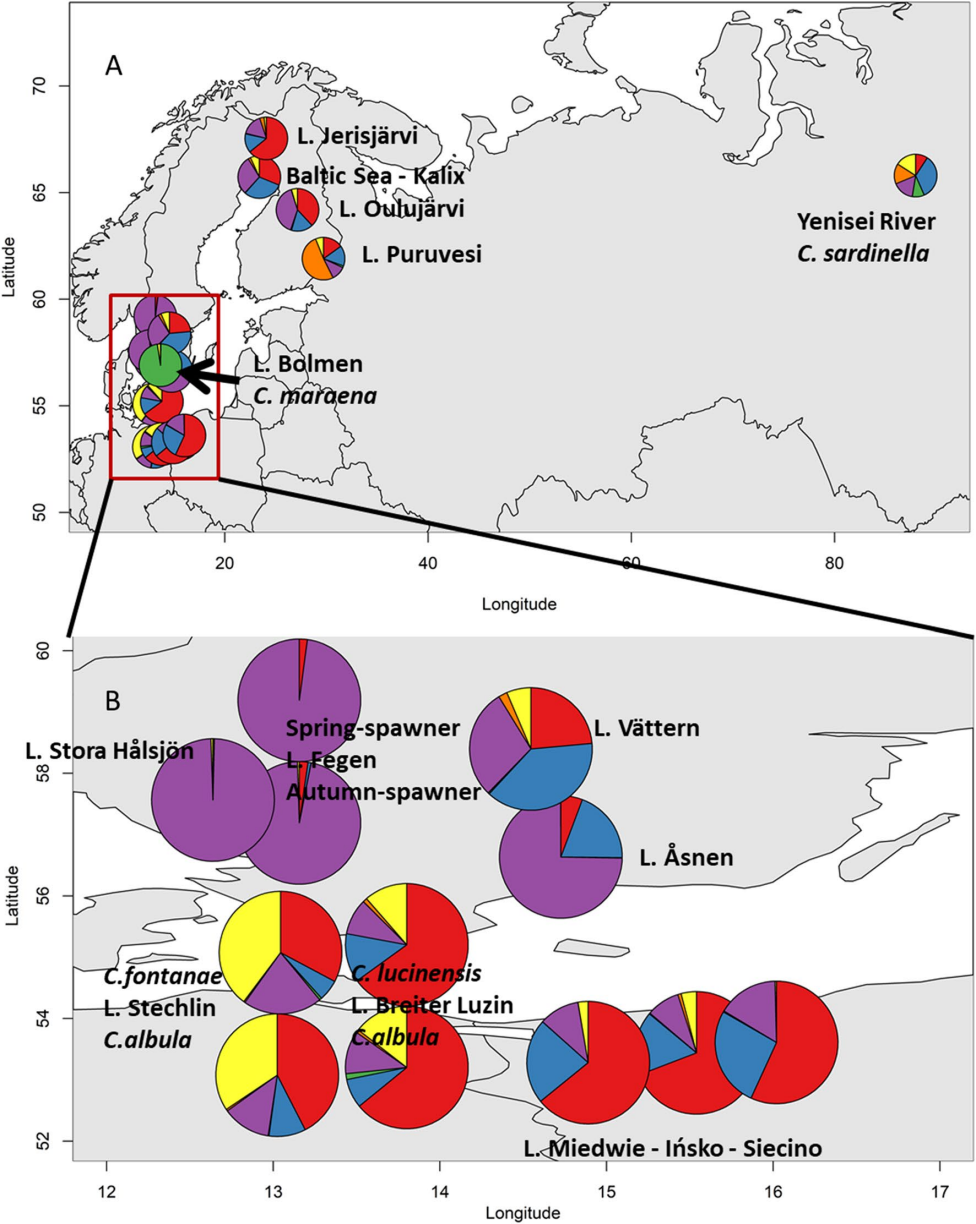
Average admixture probabilities per population. Average admixture probabilities for 18 populations of *Coregonus* in 15 locations of Europe (**A**), and of a subset of 12 populations in nine lakes around the Baltic Sea (**B**). The admixture coefficients for six identified ancestral genetic groups were obtained by the R package LEA

When the average share of the six genetic groups per population was plotted on a geographical map, there was some similarity between the Finnish Lakes Jerisjärvi, Oulujärvi and the Swedish Baltic Sea population near Kalix that are also geographically close to each other (Fig. 5A). In contrast, the admixture composition of the Finnish Lake Puruvesi was distinct. For the populations around the Baltic Sea, the similarity in admixture probabilities for the lakes of Swedish microsatellite assemblage I (Fegen, Stora Hålsjön and Åsnen) was obvious (Fig. 5B). In general, admixture probabilities were dissimilar among the populations north and south of the Baltic Sea (Swedish and Finnish versus German and Polish lakes). The sympatric pairs from the German Lakes Stechlin and Breiter Luzin were more similar to each other than to those in the other German lake, and Lake Breiter Luzin populations shared some admixture probabilities with the three allopatric *C. albula* from western Poland (Fig. 5B).

## Discussion

We aimed to analyze the genetic differentiation as based on neutral nuclear markers among 16 populations of Baltic ciscoes and one *C. sardinella* and one *C. maraena* population as distinct groups, which covered a broad geographic origin from northern Germany, Poland, Sweden, Finland and Siberia. The two sympatric cisco pairs in the German Lakes Stechlin and Breiter Luzin were only weakly differentiated from populations in neighboring Poland, and share some similarities with cisco populations from Finland and Sweden (microsatellite assemblage II as described in [32]), for which no sympatric pairs have been described. These similarities are indicated by the low genetic distances and pairwise θ between German, Polish and Swedish assemblage II populations. In contrast, the sympatric ciscoes in Lake Fegen and the allopatric *C. albula* populations at higher altitude in southern Swedish lakes (microsatellite assemblage I) are genetically most segregated from all other cisco populations included. Accordingly, the German and Swedish sympatric cisco pairs in Lakes Stechlin, Breiter Luzin and Fegen most likely do not share a common phylogeographic origin. Our data thus confirmed a stronger genetic similarity of sympatric cisco pairs within lakes than among the three autumn- or spring-spawners of the three lakes, suggesting independent evolution of the pairs in each of the lakes. Furthermore, we detected that the population differentiation of the sympatric cisco populations in the two German lakes, as estimated by microsatellite analyses, was very weak and seems to have decreased in recent years. Accordingly, the sympatric populations in German Lakes Stechlin and Breiter Luzin may be at risk of extinction. In contrast, the similar sympatric population pair in Lake Fegen showed strongly significant population differentiation, although the genetic difference between these sympatric populations may also be in decline [33].

The dendrogram (Fig. 2) split the 16 Baltic cisco populations essentially according to geographical origin, with three higher-order groups having relatively high (> 80%) bootstrap support; i.e. the four German combined with the three Polish populations, and the populations of the Swedish microsatellite assemblages I and II, respectively. In contrast, the positions in the dendrogram of the three Finnish populations were more uncertain (< 50% bootstrap support), despite being located in relatively close geographical proximity; *C. albula* from the Finnish lakes, *C. maraena* and *C. sardinella* were separated from the populations forming the clusters I–III. This overall grouping of the populations was confirmed by the Principal Coordinate Analysis.

In line with the dendrogram and the PCoA biplots, the average admixture coefficients (Figs. 4 and 5) generally reflected geographical origin; in particular, the neighboring four German and three Polish populations shared substantial similarity with respect to putative ancestral genetic groups. In contrast, the geographically most distant populations from those in Germany and Poland were the Finnish Lakes Jerisjärvi, Oulujärvi and Puruvesi, the Swedish Baltic Sea population near Kalix and the Russian Yenisei River, which all had admixture coefficients for ancestral genetic groups not shared by the German and Polish ciscoes. However, even these populations displayed substantial overlap for the majority of admixture coefficients with the cisco populations in German, Poland and southern Sweden. These patterns suggest that the contemporary genetic differentiation between the cisco populations including *C. sardinella* from Yenisei River has evolved (and is maintained) by random genetic drift in isolated populations. However, all lakes are relatively large (> 300 ha surface area) and hence the population sizes of ciscoes can be expected to be > 100,000 individuals, likely preventing strong effects of genetic drift. The documented shared ancestry of populations in the same geographic area can be explained by gene flow in connection to post-glacial immigrations.

The strong effect of post-glacial processes on the phylogeography of the Baltic cisco populations becomes obvious if the differentiation among the Swedish populations is taken into account. Lakes of the Swedish microsatellite assemblage I, in particular Lakes Fegen and Stora Hålsjön, were exceptions from a clear geography-based differentiation. Their admixture coefficients were very homogenous, with a single dominant putative ancestral genetic group that was only weakly shared by the other populations around the Baltic Sea. Populations of the assemblage I exist in southern Swedish higher-elevation lakes, including the sympatric populations from Lake Fegen and from the three other lakes, in which only the autumn-spawning *C. albula* populations survived while the spring-spawners became extinct during the last decades (Lakes Stora Hålsjön, Asunden, Ören) [33]. Mitochondrial DNA haplotypes of this group resembled those in North American *C. sardinella* populations, suggesting an immigration wave connecting these populations.

In contrast, the *C. sardinella* population from the Siberian Yenisei River in our study was more similar to Swedish assemblage II than to assemblage I (Fig. 2), with pairwise θ being substantially lower between the Yenisei and Lake Vättern and Kalix ciscoes than between Yenisei and the other Swedish populations (Additional file 1: Table S5). This pattern suggests that during the last glaciation of Europe there has been a second refugial area in the east, which connects genetically the present Siberian *C. sardinella* populations with the Swedish *C. albula* in assemblage II.

The post-glacial colonization of central and north Europe from an eastern refugial area is also supported by the closer clustering of the Finnish *C. albula* population in Lake Puruvesi with the Siberian *C. sardinella* (although with bootstrap support < 50%), and by the fact that the Finnish, Polish and German populations together were more distant to the Swedish assemblage I than to assemblage II. It is further possible that the present populations in Germany and perhaps Poland are related to both colonization waves and are even connected to Siberian and North American *C. sardinella* as indicated by the presence of both mtDNA clades in Breiter Luzin [34]. However, regarding the populations from Finnish lakes and of *C. sardinella*, it has to be admitted that the results were most impacted by missing microsatellite alleles in our study, and hence the number of analyzed individuals was lower than for most of the other populations, likely reflected as low bootstrap supports in the dendrogram.

Recent studies comparing Baltic and White Sea (Russia) *C. albula* and Siberian *C. sardinella* populations suggested that all contemporary populations in this area were formed by post-glacial hybridization between locally surviving ciscoes and invading West or East Siberian *C. sardinella* [39, 40]. Similar phylogeographic patterns have been found for the cold-water gadoid fish species burbot (*Lota lota*) for which populations from around the Baltic Sea formed a mitochondrial clade together with populations from Siberia, with single haplotypes even shared with burbot populations from Beringia and Alaska [41]. In contrast, the phylogeography of whitefishes (*Coregonus* spp.) [17], brown trout (*Salmo trutta*) [42] and Eurasian perch (*Perca fluviatilis*) [43] indicated a stronger post-glacial re-invasion of central and north European locations from southern or south-eastern refugial areas, which may have been inhabitable for the cold-water ciscoes and burbot.

The microsatellite analyses of this study support the hypothesis that the three sympatric *Coregonus* cisco pairs in Swedish and German lakes evolved independently in each of the lakes, forming another example of parallel speciation, similar to the patterns found for example in other fish species [44–46], lizards [47] or snails [48]. If the evolutionary origin of the autumn- and spring-spawners had been two different species, we would have expected that all spring-spawners form a distinct group, while the autumn-spawners would group with the allopatric *C. albula* populations in the other lakes. The higher genetic similarity within than among the lakes supports earlier studies on these species pairs in German lakes based on microsatellites and mtDNA [34], and on similar studies in Swedish lakes [32]. A potential explanation of independent evolution is lake-specific post-glacial sympatric speciation, as for example likewise suggested for the Finnish sympatric autumn- and winter-spawning ciscoes [31], and for sympatric *C. artedi* forms in the North-American Great Lakes area [49]. In addition to the substantially differing spawning times, the sympatric populations differ morphologically and in growth rates [27–29, 33, 50, 51].

More detailed investigations on ecological segregation have been performed on the sympatric pair in Lake Stechlin. A potential evolutionary split from a single ancestral population along the vertical temperature gradient has been demonstrated by an adaptive-dynamics model [52]. As a consequence of ecological divergence, the autumn-spawning *C. albula* and the spring-spawning *C. fontanae* in Lake Stechlin differ in their final thermal preferendum [53], in the optimum temperature for swimming [19] and in their temperature-related vertical distribution [54]. The spring-spawners prefer colder temperatures and occur in deeper and colder waters than the autumn-spawners. Similar adaptations to differing temperatures have been reported for sibling species in scorpionflies [55] or polychaete worms [56]. However, there is a surprisingly weak ecological differentiation between the sympatric ciscoes with respect to diet composition [57] and feeding efficiency at differing prey densities and illumination strengths [58], suggesting that the populations did not diverge along the pelagic-benthic feeding niche, as often observed for different whitefishes or Arctic char [13, 16]. We do not know whether similar patterns also differentiate the sympatric cisco pairs in Lakes Breiter Luzin or Fegen because the ecology and physiology of these pairs is only poorly studied. However, at least both German lakes have large hypolimnetic volumes, indicated by maximum lake depths that are bigger than those of most of the other lakes in northern Germany (Lake Stechlin: 69 m, Lake Breiter Luzin: 58 m) (compare [59]) that would make it plausible that ecological segregation along vertical temperature gradients may have shaped the evolution of both German sympatric cisco pairs.

The statistically non-significant genetic differentiation between the sympatric pair in Lake Breiter Luzin and the very low differentiation in Lake Stechlin were not unexpected. Previous results on population differentiation of the German sympatric pairs [34, 35] had demonstrated statistically significant F_ST_ estimates for samples taken in the years before 2005, but the differentiation was already weak. In the recent study, we could further follow up the genetic differentiation of these populations within lakes over time as based on repeated catches between 2011 and 2018. There was a tendency that genetic structure within the *C. albula* population in Lake Stechlin differed between 2011 and 2018, but we did not find evidence for a systematic differentiation of populations between all sampling years. Similar weak genetic differentiation has earlier been reported for the now extinct sympatric populations in Swedish Lake Ören [60, 61]. In contrast, and as observed earlier, the pairwise θ was much higher and strongly significant between the similar pair of autumn- and spring-spawning cisco populations in Swedish Lake Fegen [32, 33].

The spawning period (autumn: November to December vs. spring: April to June) of both sympatric pairs in the German lakes is still distinct, and hence reproductive isolation can be expected to be functionally maintained. Based on the available datasets of nine microsatellite markers, we have not evaluated the amount of potential hybridization between the species pairs. In the earlier study based on the numerically more abundant AFLP-markers, however, there were some signs of hybridization between the sympatric populations in both lakes, documented for fishes caught around the year 2000 [35]. We cannot exclude that the hybridization rate has increased, and hence the genetic differentiation between the sympatric species has weakened further during the last decades. This would mean that gene flow between the populations may have increased, potentially caused by spring-spawning individuals that reach maturity much earlier (winter) due to eutrophication-supported faster growth. However, we have never observed ripe individuals of both species simultaneously, but would not fully exclude that a short overlap in spawning season has developed due to eutrophication. Alternatively, stochastic drift may contribute to slightly differing degrees of population differentiation between years. *Coregonus albula* populations are well-known for abundance cycles with strongly varying recruitment [38, 62] that may affect also the genetic structure of the populations between the years.

There are three potential explanations why the genetic differentiation between the German sympatric pairs is much lower than that of the sympatric pair in Lake Fegen. First, it is possible that the population split between the sympatric populations from a common ancestor has occurred earlier in the Swedish than in the German populations, and hence the population differentiation is not yet strongly developed in Lakes Stechlin and Breiter Luzin. For Lake Fegen, time since divergence has been estimated to around 1900 years only (95% p.i.: 350 to 5900 years) assuming a generation interval of 4 years [33], a surprisingly short time frame to be tested in the future. According to the weaker differentiation in the German lakes, the time since divergence in Lakes Stechlin and Breiter Luzin may have been even shorter. Second, both Lake Stechlin and Lake Breiter Luzin have suffered from strong eutrophication during the last decades, resulting in a gradual loss of cold and oxygenated deep-water habitats [36–38]. For the Lake Stechlin ciscoes, physiological segregation along the vertical temperature profile was considered the major speciation route along which the sympatric pair may have evolved [19, 52]. Therefore, we cannot exclude that the recent eutrophication of both lakes has contributed to weakening the genetic differentiation. A loss of sympatric species and decline of genetic variability in response to eutrophication are well documented also for subalpine whitefish populations [23, 63] or North-American *C. clupeaformis* [64]. In other cases, fish stocking, water level regulations or species invasion induced similar reductions of reproductive isolation between sympatric *Coregonus* populations [65, 66]. A recent detailed study on the sympatric populations in Lake Fegen revealed that a potential increase in gene flow from autumn‐spawners to spring-spawners could have resulted in a change towards a less distinct phenotype in spring‐spawners [33]. Accordingly, changing environmental conditions since the last glaciation may have induced evolution and disappearance of several sympatric pairs of ciscoes. It is remarkable that three out of four sympatric pairs from Sweden have disappeared during the last decades. It cannot be excluded that there is a major risk that hybridization and change in habitat quality massively threaten the long-term population existence also of the sympatric pairs in the two German lakes, in particular because the genetic differentiation is only weakly expressed in our microsatellite dataset.

Third, phenotypic traits may be affected by few genetic major effect loci only, but their ecological functions may induce strong additional effects on assortative mating and hence couple pre- and post-zygotic isolation. A recent study on Midas cichlids (*Amphilophus* spp.) demonstrated that such mono- or oligogenic traits may not suffice to efficiently reducing gene flow, such that genome-wide differentiation can remain weak and hence distinct species are ultimately not formed [67]. If the physiological traits of the sympatric ciscoes in Lake Stechlin are affected by few major effect loci only, it would even be possible that the speciation process was mainly driven by secondary contacts between ancestors from different glacial refugial areas, and the genetic differentiation was lost from gene flow, apart from genomic islands of differentiation. Such secondary divergence has been identified as the major process in the evolution of sympatric populations of whitefish (*Coregonus* spp.) in lakes of Switzerland and Norway [68]. High-resolution marker sets, for example based on Single Nucleotide Polymorphisms (SNPs) obtained by RAD-sequencing (e.g., [69]), or whole-genome sequencing would be valuable approaches to decipher the current status and the speciation history of the sympatric populations in Lakes Stechlin, Breiter Luzin and Fegen.

The complex phylogeographic history and the low differentiation at neutral markers in the German sympatric populations challenge the current taxonomy of sympatric Baltic ciscoes. Similar to the taxonomy of *Coregonus* whitefishes, also sympatric Baltic ciscoes are considered distinct species in Central Europe, whereas they are considered the same species in Scandinavia [15]. The different taxonomy may be rooted in history (*C. lucinensis* was first described already in 1933), and also involve different taxonomical traditions in various parts of Europe. However, as discussed before, the level of genetic differentiation alone is not indicative to discriminate between complete and incomplete speciation. Non-overlapping spawning times, as found for the sympatric Baltic ciscoes, may be sufficient to limit gene flow between the co-occurring populations with differentiation accumulating over longer time spans, and hence may be seen as a central element to consider spring-spawning ciscoes as separate species. However, we do not know enough about the differences in genomic architecture of the sympatric species to evaluate their degree of post-zygotic incompatibility. As long as this ambiguity remains, the spring-spawning ciscoes in the two German lakes are considered separate evolutionary significant units, and their population status is monitored continuously.

## Conclusion

Our data suggest that for the three sympatric pairs of autumn- and spring-spawning ciscoes, a common phylogeographic history is unlikely. The sympatric ciscoes in Lake Fegen and the allopatric *C. albula* populations in other Swedish lakes of the microsatellite assemblage I are genetically most segregated from all other cisco populations included here. In contrast, the two sympatric pairs in the German Lakes Stechlin and Breiter Luzin are only weakly differentiated and most closely related to populations in neighboring Poland. The statistically non-significant neutral genetic differentiation of both German sympatric pairs in comparison with the strong genetic differentiation of the sympatric pair in Lake Fegen makes both pairs more prone to the loss of the locally endemic spring-spawners. Recent eutrophication in both lakes may add to the disturbance of strong ecological gradients that may have been decisive during the ecological split between autumn- and spring-spawners. In the complex of Baltic ciscoes, disappearance of endemic spring-spawners from several locations has already been documented, presumably caused by anthropogenic effects on the lakes. These observations support the general notion that biodiversity is at risk from anthropogenic changes, such as global warming. Species such as the endemic spring-spawning ciscoes that only recently evolved may be particularly vulnerable.

## Methods

### General

We included 18 *Coregonus* populations from 15 localities into the analyses (Fig. 1). Two sympatric population pairs from German lakes were analysed, namely *C. albula* and *C. fontanae* from Lake Stechlin, and *C. albula* and *C. lucinensis* from Lake Breiter Luzin, Fig. 1B). We added the sympatric autumn-spawning and spring-spawning populations from Swedish Lake Fegen. Furthermore, four allopatric *C. albula* populations from Swedish lakes were added, with two each belonging to the previously identified Swedish microsatellite assemblage I (Lakes Stora Hålsjön, Åsnen) or assemblage II (Lake Vättern, and a population from the Gulf of Bothnia, Baltic Sea, near the village of Kalix) [32] (Fig. 1A, B). We did not consider all Swedish populations from the previous complete analysis [32], because our aim was to identify potential genetic similarity of the newly included populations from Germany, Finland, Poland and Russia to the two Swedish assemblages, but not to every single lake within the Swedish assemblages. Three *C. albula* populations from western Polish Lakes Ińsko, Siecino and Miedwie, and three populations from the Finnish Lakes Jerisjärvi, Oulujärvi and Puruvesi were also added. Finally, we obtained individuals from a population of the Russian Yenisei River near Turukhansk, which is preliminarly identified as *C. sardinella*. For comparison, we also considered a population of *C. maraena* (whitefish) from the Swedish Lake Bolmen as a more distantly related taxon.

Individuals from the German sympatric populations were sampled by gillnetting during the spawning times of the respective populations (late autumn: both *C. albula*, spring: *C. fontanae* and *C. lucinensis*) in the years between 2011 and 2018. We only included individuals with almost running gonads, to guarantee that we do not mix specimens from the sympatric populations. *C. albula* from Lake Stechlin was sampled in 2011, 2012, 2014 and 2018. *C. fontanae* (Lake Stechlin) was sampled in 2011, 2012 and 2018. Lake Breiter Luzin ciscoes were sampled in 2011 (both species) and 2012 (only *C. albula*). Samples from the other seven populations were obtained by gillnetting in 2002 (Finland), 2012 (Poland) and 2013 (Russia). One pectoral or caudal fin per fish was clipped and instantaneously fixed in ethanol for genetic analyses. Material from Swedish populations wasalso obtained by gillnetting and sampled for tissue in 2005, 2007, 2008, 2009 and 2010. Sympatric populations in Lake Fegen were sampled during spawning seasons, including only specimens with running or almost running gonads. See [32] for details.

### Microsatellite analysis

We conducted the laboratory analyses for the 11 populations from Germany, Poland, Finland and Russia, whereas already existing microsatellite data from the 7 Swedish populations were added later to the dataset (see below). Total DNA was extracted from fin tissue using the Tissue DNA Mini Kit peqGOLD (VWR PeqLab, Darmstadt, Germany) following the protocol of the supplier. DNA concentration was determined spectrographically and aliquots diluted to a final concentration of ~ 20 ng/µl. All individuals were genotyped at nine polymorphic microsatellites following the protocol developed by [32], namely at *BWF1*, *BWF2* [70], *Cisco90*, *Cisco126*, *Cisco157* [18], *Cocl23* [71], *Sfo8*, *Sfo23* [72], and *Str73* [73]. The three multiplex sets, i.e. simultaneous PCR amplification of multiple markers, were composed of the following loci (fluorescent dyes in brackets; Biomers, Ulm, Germany): set 1 consisted of *Cisco90* (BMN-6), *Sfo23* (Cyanine 5), *Sfo8* (Dy-751) and *Cocl23* (BMN-6), set 2 was composed of *Cisco157* (Dy-751), *Cisco126* (BMN-6) and *BWF1* (Cyanine 5), and set 3 consisted of *Str73* (BMN-6) and *BWF2* (Cyanine 5).

The Qiagen Multiplex PCR kit was used for PCR amplification according to the recommendations of the manufacturer. PCR amplification was carried out in 10 µl reaction volumes, containing 5 µl QIAGEN^®^ Multiplex PCR mastermix, 1 µl DNA (~ 20 ng/µl), 0.44 to 1.91 µl primer mix and 2.1 to 3.6 µl H_2_O (PCR grade). The thermocycling profile started with an initial denaturation step at 95 °C for 5 min, followed by 28 cycles of 30 s at 95 °C, 90 s at 53 °C, 45 s at 72 °C and ended with a final extension step of 20 min at 60 °C. A quantity of 0.85 μl of the PCR product were added to a mix of 30 μl formamide (SLS; Sciex, Darmstadt, Germany) and 1 μl size standard-400 (Sciex), and denatured fragments were resolved on an automated DNA sequencer (Beckmann Coulter CEQ 8000). Genotypes were determined with the fragment analysis module of the GenomeLab™ GeXP Genetic Analysis System, version 10.2 (Beckman Coulter). Analyses of individuals with missing genotypes (at one or several markers) were repeated once or twice, but were left incomplete after the second repetition.

To facilitate the comparison of population structure as obtained for the 11 analysed populations with that determined earlier for *C. albula* populations from Swedish lakes [32], we re-analysed 14 individuals from six Swedish populations on our sequencer. For eight of the individuals (two each from Lakes Fegen, Stora Hålsjön and Siljan, one each from Lakes Åsnen and Mälaren), we could successfully repeat results for all nine loci. As expected for microsatellite data produced at different laboratories, there were systematic differences in the number of repeats per locus (2–6 more on our sequencer, Additional file 1: Table S6). To make the newly created dataset comparable with the earlier data from Swedish lakes, we added the calculated difference in read length (Additional file 1: Table S6) to each locus for *C. albula* individuals from Lakes Fegen (autumn- and spring-spawners), Stora Hålsjön, Åsnen, Vättern, from the Baltic Sea (Kalix) population of *C. albula*, and for *C. maraena* from Lake Bolmen. In this way, the data from Swedish populations were expressed with similar read lengths as if they would have been analyzed entirely on our sequencer.

### Statistical data analyses

Most analysis and graphical output were performed in R version 3.6.2 [74]. Genetic diversity was quantified as number of alleles per population and allelic richness A_R_, i.e. the allele number corrected for the smallest sample size (N = 10) [75], by PopGenReport 3.0 [76]. Allele frequency-based correlations (inbreeding coefficient F_IS_) and population-wide deviations from Hardy–Weinberg equilibrium (HWE) were tested by the exact (probability) test using genepop_in_R based on Genepop 4.7.0 [77, 78]. The probabilities for locus-specific deviations from HWE [79] per population were corrected by the false discovery rate for multiple tests [80], completed by U-tests on excess of homozygotes. To contrast within- and between-population variance for the 18 populations, we ran an Analysis of Molecular Variance (AMOVA) [81] as implemented in the R-package pegas [82].

Population differentiation was estimated by F-statistics [83] between populations (pairwise θ), with significance of differentiation assessed through exact conditional contingency-table tests for genotypic differentiation, both performed by Genepop. To detect genetic relationships among all 18 *Coregonus* populations, a neighbor-joining tree [84] based on pairwise Cavalli-Sforza and Edwards [85] chord distance was constructed using PHYLIP [86]. This tree was used to visualize the potential similarity of our results with those obtained earlier from the entire dataset of Swedish cisco populations using the same method [32]. In addition, we ran a Principal Coordinate Analysis (PCoA), equivalent to metric multidimensional scaling, on the 655 individual genotypes, to visualize the distribution of individuals and population centroids in reduced space. This analysis was performed in ade4 [87], and the result graphs plotted by adegraphics [88].

To estimate and visualize population genetic structure, Bayesian computer algorithms as implemented in the stand-alone programs STRUCTURE [89] or ADMIXTURE [90] are typically used. To keep the analytical workflow within R, we instead applied the R-package LEA [91, 92] that provides the same functions as STRUCTURE. Similar to Bayesian clustering programs, LEA includes an R function to estimate individual admixture coefficients from the genotypic matrix [89]. Assuming K ancestral genetic groups, the R function snmf provides least-squares estimates of ancestry proportions [93]. The snmf function also estimates an entropy criterion that evaluates the quality of fit of the statistical model to the data using a cross-validation technique. The entropy criterion that is similarly implemented in LEA as in ADMIXTURE can help choosing the number of ancestral genetic groups that best explains the genotypic data [90, 93]. We run the procedure to find the smallest value of *K* for N = 1 to 15 ancestral genetic groups with 10 repetitions per *K*, the maximum number being similar to the number of sampled locations. We then ran the ancestry estimates for the 655 individuals with the optimum *K*, and displayed the coefficients as barplot. We re-run an Analysis of Molecular Variance with the optimum number of ancestry groups determined by LEA, and compared the results with those from the AMOVA on the 18 contemporary populations. Finally, we calculated the population means of the ancestry coefficients, and displayed them as pie charts on geographical maps, to illustrate the geographical distribution of the ancestry genetic groups.

## Supplementary Information

**Additional file 1: Table S1.** Proportion of missing genotypes among the nine microsatellites (columns) in the 18 *Coregonus* populations (rows). Individuals with more than a single missing locus were omitted from subsequent statistical analyses. AS = autumn-spawner, SS = spring-Spawner. **Table S2.** Matrix of pairwise θ between sympatric populations of Lake Stechlin (*Coregonus albula* and *C. fontanae*), sampled in different years (below diagonal), and their lower 95% confidence intervals (above diagonal). Weak structure between (sub)populations is indicated by the lower CI including zero. **Table S3.** Matrix of pairwise θ between sympatric populations of Lake Breiter Luzin (*Coregonus albula* and *C. lucinensis*), sampled in different years (below diagonal), and their lower 95% confidence intervals (above diagonal). Weak structure between (sub)populations is indicated by the lower CI including zero. **Table S4.** P-values of deviations from Hardy–Weinberg equilibrium per locus and population, with P < 0.05 considered a significant deviation. The original alpha = 0.05 was controlled by the false discovery rate for multiple tests. **Table S5.** Matrix of pairwise θ between 18 sympatric and allopatric *Coregonus* populations from 15 lakes or rivers in Germany, Sweden, Poland, Finland and Russia (below diagonal), and their P-values as obtained by G-tests (above diagonal). Strong structure between populations is indicated in bold. AS = autumn-spawner, SS = spring-spawner. **Table S6.** Overview of systematic differences in repeat length of nine bi-allelic microsatellite markers for eight cisco individuals from six Swedish populations, analysed either on an ABI3130xl sequencer (Applied Biosystems) (SWE) or an Beckmann Coulter CEQ 8000 sequencer (GER). The differences in read length were added to the original data for the Swedish populations included in the study. **Figure S1.** Overview on number of alleles per nine microsatellite loci, for 655 individuals of Baltic ciscoes from 18 lake and river populations in Germany, Sweden, Poland, Finland and Russia. **Figure S2.** Separate biplots of 18 *Coregonus* populations in reduced space along axes 1 and 2 of the Principal Coordinate Analysis. The axes dimensions are identically scaled to facilitate comparison of location and extent of population diversification. **Figure S3.** Plot of cross-entropy vs. a range of 1 to 15 ancestral genetic groups, as estimated by cross-validation by the R-package LEA.

## Data Availability

The microsatellite data of this study are accessible at Dryad via 10.5061/dryad.18931zczc.

## Abbreviations

AFPL: Amplified Fragment Length Polymorphism
AMOVA: Analysis of molecular variance
A_R_: Allelic richness, the mean number of alleles per locus and population adjusted to the minimum number of individuals genotyped in any population
AS: Autumn-spawner
CI: Confidence interval
F_IS_: Inbreeding coefficient
HWE: Hardy–Weinberg equilibrium
mtDNA: Mitochondrial deoxyribonucleic acid
PCR: Polymerase chain reaction
RAD: Restriction site associated deoxyribonucleic acid
SNP: Single Nucleotide Polymorphism
SS: Spring-spawner

## References

[1] Butlin RK, Galindo J, Grahame JW (2008). Sympatric, parapatric or allopatric: the most important way to classify speciation?. Philos Trans R Soc B.

[2] Dieckmann U, Tautz D, Doebeli M, Metz JAJ, Dieckmann U, Tautz D, Doebeli M, Metz JAJ (2004). Epilogue. Adaptive speciation.

[3] Hendry AP (2009). Ecological speciation! Or the lack thereof?. Can J Fish Aquat Sci.

[4] Bolnick DI, Fitzpatrick BM (2007). Sympatric speciation: models and empirical evidence. Annu Rev Ecol Evol Syst.

[5] Via S (2001). Sympatric speciation in animals: the ugly duckling grows up. Trends Ecol Evol.

[6] Nosil P, Egan SP, Funk DJ (2008). Heterogeneous genomic differentiation between walking-stick ecotypes: "Isolation by adaptation" and multiple roles for divergent selection. Evolution.

[7] Ogden R, Thorpe RS (2002). Molecular evidence for ecological speciation in tropical habitats. Proc Natl Acad Sci USA.

[8] Huber SK, De Leon LF, Hendry AP, Bermingham E, Podos J (2007). Reproductive isolation of sympatric morphs in a population of Darwin's finches. Proc R Soc B-Biol Sci.

[9] Barluenga M, Stölting KN, Salzburger W, Muschick M, Meyer A (2006). Sympatric speciation in Nicaraguan crater lake cichlid fish. Nature.

[10] Bernardi G (2013). Speciation in fishes. Mol Ecol.

[11] Schluter D (1996). Ecological speciation in postglacial fishes. Philos Trans R Soc B.

[12] Taylor EB, Bentzen P (1993). Evidence for multiple origins and sympatric divergence of trophic ecotypes of smelt (*Osmerus*) in northeastern North-America. Evolution.

[13] Jonsson B, Jonsson N (2001). Polymorphism and speciation in Arctic charr. J Fish Biol.

[14] Schluter D, McPhail JD (1993). Character displacement and replicate adaptive radiation. Trends Ecol Evol.

[15] Kottelat M, Freyhof J (2007). Handbook of European freshwater fishes.

[16] Hudson AG, Vonlanthen P, Müller R, Seehausen O (2007). Review: the geography of speciation and adaptive radiation in coregonines. Archiv für Hydrobiologie Special Issues Adv Limnol.

[17] Ostbye K, Bernatchez L, Naesje TF, Himberg KJM, Hindar K (2005). Evolutionary history of the European whitefish *Coregonus lavaretus* (L.) species complex as inferred from mtDNA phylogeography and gill-raker numbers. Mol Ecol.

[18] Turgeon J, Estoup A, Bernatchez L (1999). Species flock in the North American Great Lakes: molecular ecology of lake Nipigon Ciscoes (Teleostei: Coregonidae: *Coregonus*). Evolution.

[19] Ohlberger J, Mehner T, Staaks G, Hölker F (2008). Temperature-related physiological adaptations promote ecological divergence in a sympatric species pair of temperate freshwater fish *Coregonus* spp. Funct Ecol.

[20] Ohlund G, Bodin M, Nilsson KA, Ohlund SO, Mobley KB, Hudson AG, Peedu M, Brannstrom A, Bartels P, Praebel K (2020). Ecological speciation in European whitefish is driven by a large-gaped predator. Evol Lett.

[21] Johannesson K (2001). Parallel speciation: a key to sympatric divergence. Trends Ecol Evol.

[22] Hudson AG, Vonlanthen P, Bezault E, Seehausen O (2013). Genomic signatures of relaxed disruptive selection associated with speciation reversal in whitefish. BMC Evol Biol.

[23] Vonlanthen P, Bittner D, Hudson AG, Young KA, Muller R, Lundsgaard-Hansen B, Roy D, Di Piazza S, Largiader CR, Seehausen O (2012). Eutrophication causes speciation reversal in whitefish adaptive radiations. Nature.

[24] Seehausen O, vanAlphen JJM, Witte F (1997). Cichlid fish diversity threatened by eutrophication that curbs sexual selection. Science.

[25] Taylor EB, Boughman JW, Groenenboom M, Sniatynski M, Schluter D, Gow JL (2006). Speciation in reverse: morphological and genetic evidence of the collapse of a three-spined stickleback (*Gasterosteus aculeatus*) species pair. Mol Ecol.

[26] Hasselman DJ, Argo EE, Mcbride MC, Bentzen P, Schultz TF, Perez-Umphrey AA, Palkovacs EP (2014). Human disturbance causes the formation of a hybrid swarm between two naturally sympatric fish species. Mol Ecol.

[27] Thienemann A (1933). *Coregonus albula lucinensis*, eine Tiefenform der Kleinen Maräne aus einem norddeutschen See. Zugleich ein Beitrag zur Rassenbildung bei *Coregonus albula* L. Z Morph Ökol Tiere.

[28] Svärdson G. Speciation of the Scandinavian *Coregonus* reports of the institute of freshwater research, Drottningholm. 1979; 57:1–95.

[29] Schulz M, Freyhof J (2003). *Coregonus fontanae*, a new spring-spawning cisco from Lake Stechlin, northern Germany (Salmoniformes: Coregonidae). Ichthyol Explor Freshw.

[30] Airaksinen KJ (1968). Preliminary notes on the winter-spawning vendace (*Coregonus albula* L.) in some Finnish lakes. Ann Zool Fenn.

[31] Vuorinen J, Himberg MKJ, Lankinen P (1981). Genetic differentiation in *Coregonus albula* (L.) (Salmonidae) populations in Finland. Hereditas.

[32] Delling B, Palm S, Palkopoulou E, Prestegaard T (2014). Genetic signs of multiple colonization events in Baltic ciscoes with radiation into sympatric spring- and autumn-spawners confined to early postglacial arrival. Ecol Evol.

[33] Delling B, Palm S (2019). Evolution and disappearance of sympatric *Coregonus albula* in a changing environment-a case study of the only remaining population pair in Sweden. Ecol Evol.

[34] Schulz M, Freyhof J, Saint-Laurent R, Østbye K, Mehner T, Bernatchez L (2006). Evidence for independent origin of two spring-spawning ciscoes in Germany (Salmoniformes: Coregonidae). J Fish Biol.

[35] Mehner T, Pohlmann K, Elkin C, Monaghan MT, Nitz B, Freyhof J (2010). Genetic population structure of sympatric and allopatric populations of Baltic ciscoes (*Coregonus albula* complex, Teleostei, Coregonidae). BMC Evol Biol.

[36] Koschel R, Benndorf J, Proft G, Recknagel F (1983). Calcite precipitation as a natural control mechanism of eutrophication. Arch Hydrobiol.

[37] Selmeczy GB, Abonyi A, Krienitz L, Kasprzak P, Casper P, Telcs A, Somogyvári Z, Padisák J (2019). Old sins have long shadows: climate change weakens efficiency of trophic coupling of phyto- and zooplankton in a deep oligo-mesotrophic lowland lake (Stechlin, Germany)—a causality analysis. Hydrobiologia.

[38] Braun LM, Brucet S, Mehner T (2021). Top-down and bottom-up effects on zooplankton size distribution in a deep stratified lake. Aquat Ecol.

[39] Borovikova EA, Artamonova VS (2021). Vendace (*Coregonus albula*) and least cisco (*Coregonus sardinella*) are a single species: evidence from revised data on mitochondrial and nuclear DNA polymorphism. Hydrobiologia.

[40] Sendek DS (2021). Phylogenetic relationships in vendace and least cisco, and their distribution areas in western Eurasia. Ann Zool Fenn.

[41] Van Houdt JKJ, De Cleyn L, Perretti A, Volckaert FAM (2005). A mitogenic view on the evolutionary history of the Holarctic freshwater gadoid, burbot (*Lota lota*). Mol Ecol.

[42] Bernatchez L (2001). The evolutionary history of brown trout (*Salmo trutta* L.) inferred from phylogeographic, nested clade, and mismatch analyses of mitochondrial DNA variation. Evolution.

[43] Nesbo CL, Fossheim T, Vollestad LA, Jakobsen KS (1999). Genetic divergence and phylogeographic relationships among European perch (*Perca fluviatilis*) populations reflect glacial refugia and postglacial colonization. Mol Ecol.

[44] Palkovacs EP, Dion KB, Post DM, Caccone A (2008). Independent evolutionary origins of landlocked alewife populations and rapid parallel evolution of phenotypic traits. Mol Ecol.

[45] Hudson AG, Vonlanthen P, Seehausen O (2011). Rapid parallel adaptive radiations from a single hybridogenic ancestral population. Proc R Soc B-Biol Sci.

[46] Rundle HD, Nagel L, Boughman JW, Schluter D (2000). Natural selection and parallel speciation in sympatric sticklebacks. Science.

[47] Richmond JQ, Reeder TW (2002). Evidence for parallel ecological speciation in scincid lizards of the *Eumeces skiltonianus* species group (Squamata: Scincidae). Evolution.

[48] Johannesson K, Panova M, Kemppainen P, Andre C, Rolan-Alvarez E, Butlin RK (2010). Repeated evolution of reproductive isolation in a marine snail: unveiling mechanisms of speciation. Philos Trans R Soc B.

[49] Piette-Lauzire G, Bell AH, Ridgway MS, Turgeon J (2019). Evolution and diversity of two cisco forms in an outlet of glacial Lake Algonquin. Ecol Evol.

[50] Helland IP, Vøllestad LA, Freyhof J, Mehner T (2009). Morphological differences between two ecologically similar sympatric fishes. J Fish Biol.

[51] Lessmark O. Undersökningar av fiskfaunan i Fegen och Kalvsjön med speciell inriktning på de vår‐ och höstlekande siklöjorna. Information från Sötvattenslaboratoriet Drottningholm 1976:15.

[52] Ohlberger J, Brannstrom A, Dieckmann U (2013). Adaptive phenotypic diversification along a temperature-depth gradient. Am Nat.

[53] Ohlberger J, Staaks G, Petzoldt T, Mehner T, Hölker F (2008). Physiological specialization by thermal adaptation drives ecological divergence in a sympatric fish species pair. Evol Ecol Res.

[54] Mehner T, Busch S, Helland IP, Emmrich M, Freyhof J (2010). Temperature-related nocturnal vertical segregation of coexisting coregonids. Ecol Freshw Fish.

[55] Sauer KP, Vermeulen A, Aumann N (2003). Temperature-dependent competition hierarchy: a mechanism stabilizing the phenological strategy in the scorpionfly *Panorpa communis* L. J Zool Syst Evol Res.

[56] Kruse I, Strasser M, Thiermann F (2004). The role of ecological divergence in speciation between intertidal and subtidal *Scoloplos armiger* (Polychaeta, Orbiniidae). J Sea Res.

[57] Helland IP, Harrod C, Freyhof J, Mehner T (2008). Coexistence of a pair of pelagic planktivorous coregonid fish. Evol Ecol Res.

[58] Ohlberger J, Mehner T, Staaks G, Hölker F (2008). Is ecological segregation in a sympatric species pair of coregonines supported by divergent feeding efficiencies?. Can J Fish Aquat Sci.

[59] Mehner T, Holmgren K, Lauridsen TL, Jeppesen E, Diekmann M (2007). Lake depth and geographical position modify lake fish assemblages of the European 'Central Plains' ecoregion. Freshw Biol.

[60] Svärdson G (1988). Pleistocene age of the spring-spawning cisco, *Coregonus trybomi*. Nord J Freshw Res.

[61] Vuorinen J (1988). Enzyme genes as interspecific hybridization probes in Coregoninae fishes. Finn Fish Res.

[62] Hamrin SF, Persson L (1986). Asymmetrical competition between age classes as a factor causing population oscillations in an obligate planktivorous fish species. Oikos.

[63] Feulner PGD, Seehausen O (2019). Genomic insights into the vulnerability of sympatric whitefish species flocks. Mol Ecol.

[64] Reid SM, Parna M, Reist JD (2017). Collapse of Lake Whitefish *Coregonus clupeaformis* (Mitchill, 1818) species pair in Como Lake, Ontario. J Appl Ichthyol.

[65] Huuskonen H, Shikano T, Mehtatalo L, Kettunen J, Eronen R, Toiviainen A, Kekaelaeinen J (2017). Anthropogenic environmental changes induce introgression in sympatric whitefish ecotypes. Biol J Linn Soc.

[66] Bhat S, Amundsen PA, Knudsen R, Gjelland KO, Fevolden SE, Bernatchez L, Praebel K (2014). Speciation reversal in European whitefish (*Coregonus lavaretus* (L.)) caused by competitor invasion. PLoS ONE.

[67] Kautt AF, Kratochwil CF, Nater A, Machado-Schiaffino G, Olave M, Henning F, Torres-Dowdall J, Harer A, Hulsey CD, Franchini P (2020). Contrasting signatures of genomic divergence during sympatric speciation. Nature.

[68] Rougeux C, Gagnaire PA, Bernatchez L (2019). Model-based demographic inference of introgression history in European whitefish species pairs. J Evol Biol.

[69] Ackiss AS, Larson WA, Stott W (2020). Genotyping-by-sequencing illuminates high levels of divergence among sympatric forms of coregonines in the Laurentian Great Lakes. Evol Appl.

[70] Patton JC, Gallaway RG, Fechhelm RG, Cronin MA (1997). Genetic variation of microsatellite and mitochondrial DNA markers in broad whitefish (*Coregonus nasus*) in the Colville and Sagavanirkrtok Rivers in northern Alaska. Can J Fish Aquat Sci.

[71] Bernatchez L, Vuorinen JA, Bodaly RA, Dodson JJ (1996). Genetic evidence for reproductive isolation and multiple origins of sympatric trophic ecotypes of whitefish (*Coregonus*). Evolution.

[72] Angers B, Bernatchez L, Angers A, Desgroseillers L (1995). Specific microsatellite loci for brook charr reveal strong population subdivision on a microgeographic scale. J Fish Biol.

[73] Estoup A, Presa P, Krieg F, Vaiman D, Guyomard R (1993). Ct)(N) and (Gt)(N) microsatellites—a new class of genetic markers for *Salmo trutta* L (Brown trout). Heredity.

[74] R Development Core Team (2017). R a language and environment for statistical computing.

[75] ElMousadik A, Petit RJ (1996). High level of genetic differentiation for allelic richness among populations of the argan tree [*Argania spinosa* (L) Skeels] endemic to Morocco. Theor Appl Genet.

[76] Adamack AT, Gruber B (2014). PopGenReport: simplifying basic population genetic analyses in R. Methods Ecol Evol.

[77] Raymond M, Rousset F (1995). Genepop (Version-1.2)—population genetics software for exact tests and ecumenicism. J Hered.

[78] Rousset F (2008). GENEPOP ' 007: a complete re-implementation of the GENEPOP software for Windows and Linux. Mol Ecol Resour.

[79] Guo SW, Thompson EA (1992). Performing the exact test of Hardy–Weinberg proportion for multiple alleles. Biometrics.

[80] Benjamini Y, Hochberg Y (1995). Controlling the false discovery rate—a practical and powerful approach to multiple testing. J R Stat Soc B Met.

[81] Excoffier L, Smouse PE, Quattro JM (1992). Analysis of molecular variance inferred from metric distances among DNA haplotypes—application to human mitochondrial-DNA Restriction Data. Genetics.

[82] Paradis E (2010). pegas: an R package for population genetics with an integrated-modular approach. Bioinformatics.

[83] Weir BS, Cockerham CC (1984). Estimating F-statistics for the analysis of population structure. Evolution.

[84] Saitou N, Nei M (1987). The neighbor-joining method: a new method for reconstructing phylogenetic trees. Mol Biol Evol.

[85] Cavalli-Sforza LL, Edwards AWF (1967). Phylogenetic analysis: models and estimation procedures. Evolution.

[86] Felsenstein J. PHYLIP (Phylogeny Inference Package), version 3.6. Distributed by the author: Department of Genome Sciences, University of Washington, Seattle, Washington; 2004.

[87] Thioulouse J, Dray S, Dufour A, Siberchicot A, Jombart T, Pavoine S (2018). Multivariate analysis of ecological data with ade4.

[88] Siberchicot A, Julien-Laferrière A, Dufour A-B, Thioulouse J, Dray S (2017). adegraphics: an S4 Lattice-based package for the representation of multivariate data. R J.

[89] Pritchard JK, Stephens M, Donnelly P (2000). Inference of population structure using multilocus genotype data. Genetics.

[90] Alexander DH, Lange K (2011). Enhancements to the ADMIXTURE algorithm for individual ancestry estimation. BMC Bioinform.

[91] Frichot E, Francois O (2015). LEA: an R package for landscape and ecological association studies. Methods Ecol Evol.

[92] François O. Running structure-like population genetic analyses with R. In: R Tutorials in Population Genetics*.* Grenoble: University of Grenoble-Alpes. 2016:1–9.

[93] Frichot E, Mathieu F, Trouillon T, Bouchard G, Francois O (2014). Fast and efficient estimation of individual ancestry coefficients. Genetics.

