# Supplementary material for: Genetic relationships between sympatric and allopatric Coregonus ciscoes in North and Central Europe
Source: BMC Ecol Evol. 2021 Oct 6;21:186. doi: 10.1186/s12862-021-01920-8 (PMC8496053; doi:10.1186/s12862-021-01920-8)

**Table S1**: Proportion of missing genotypes among the nine microsatellites (columns) in the 18 *Coregonus* populations (rows). Individuals with more than a single missing locus were omitted from subsequent statistical analyses. AS=autumn-spawner, SS=spring-Spawner

|  | BWF1 | BWF2 | Cisco126 | Cisco157 | Cisco90 | Cocl23 | Sfo23 | Sfo8 | Str73 | Mean |
| --- | --- | --- | --- | --- | --- | --- | --- | --- | --- | --- |
| Calbula_Stechlin | 0.045 | 0.060 | 0.015 | 0.015 | 0.015 | 0.030 | 0.030 | 0.149 | 0.015 | 0.041 |
| Cfontanae_Stechlin | 0.134 | 0.119 | 0.090 | 0.045 | 0.045 | 0.090 | 0.119 | 0.299 | 0.045 | 0.109 |
| Calbula_BLuzin | 0.138 | 0.103 | 0.138 | 0.034 | 0.034 | 0.069 | 0.138 | 0.103 | 0.034 | 0.088 |
| Clucinensis_BLuzin | 0.200 | 0.100 | 0.133 | 0.033 | 0.033 | 0.133 | 0.067 | 0.100 | 0.033 | 0.093 |
| Calbula_AS_Fegen | 0.271 | 0.000 | 0.000 | 0.000 | 0.000 | 0.000 | 0.000 | 0.000 | 0.000 | 0.030 |
| Calbula_SS_Fegen | 0.000 | 0.000 | 0.000 | 0.000 | 0.000 | 0.000 | 0.000 | 0.000 | 0.000 | 0.000 |
| Calbula_Asnen | 0.000 | 0.000 | 0.000 | 0.000 | 0.000 | 0.000 | 0.000 | 0.000 | 0.000 | 0.000 |
| Calbula_Halsjön | 0.000 | 0.000 | 0.000 | 0.000 | 0.000 | 0.000 | 0.000 | 0.000 | 0.000 | 0.000 |
| Calbula_Kalix | 0.000 | 0.000 | 0.000 | 0.000 | 0.000 | 0.000 | 0.000 | 0.000 | 0.000 | 0.000 |
| Calbula_Vättern | 0.000 | 0.000 | 0.000 | 0.000 | 0.000 | 0.000 | 0.000 | 0.000 | 0.000 | 0.000 |
| Calbula_Insko | 0.000 | 0.033 | 0.000 | 0.000 | 0.000 | 0.000 | 0.000 | 0.000 | 0.000 | 0.004 |
| Calbula_Miedwie | 0.032 | 0.000 | 0.000 | 0.000 | 0.065 | 0.000 | 0.032 | 0.065 | 0.000 | 0.022 |
| Calbula_Siecino | 0.000 | 0.000 | 0.033 | 0.000 | 0.000 | 0.000 | 0.000 | 0.000 | 0.000 | 0.004 |
| Calbula_Oulujärvi | 0.063 | 0.031 | 0.031 | 0.094 | 0.094 | 0.469 | 0.406 | 0.531 | 0.000 | 0.191 |
| Calbula_Jerisjärvi | 0.421 | 0.158 | 0.368 | 0.158 | 0.211 | 0.263 | 0.263 | 0.632 | 0.158 | 0.292 |
| Calbula_Puruvesi | 0.063 | 0.063 | 0.000 | 0.000 | 0.156 | 0.313 | 0.250 | 0.281 | 0.000 | 0.125 |
| Csardinella_YeniseiRiver | 0.067 | 0.067 | 0.067 | 0.000 | 0.000 | 0.000 | 0.267 | 0.400 | 0.000 | 0.096 |
| Cmaraena_Bolmen | 0.000 | 0.000 | 0.000 | 0.000 | 0.000 | 0.000 | 0.000 | 0.000 | 0.000 | 0.000 |
| Total | 0.078 | 0.037 | 0.036 | 0.017 | 0.028 | 0.063 | 0.067 | 0.117 | 0.013 | 0.051 |

**Table S2**: Matrix of pairwise θ between sympatric populations of Lake Stechlin (*Coregonus albula* and *C. fontanae*), sampled in different years (below diagonal), and their P-values (above diagonal).

|  | Ca_Stechlin_2011 | Ca_Stechlin_2012 | Ca_Stechlin_2014 | Ca_Stechlin_2018 | Cf_Stechlin_2011 | Cf_Stechlin_2012 | Cf_Stechlin_2018 |
| --- | --- | --- | --- | --- | --- | --- | --- |
| Ca_Stechlin_2011 |  | 0.551 | 0.088 | **0.003** | 0.453 | 0.100 | **0.012** |
| Ca_Stechlin_2012 | -0.004 |  | 0.930 | 0.951 | 0.603 | 0.664 | 0.779 |
| Ca_Stechlin_2014 | 0.002 | -0.044 |  | 0.130 | 0.232 | 0.773 | 0.229 |
| Ca_Stechlin_2018 | **0.016** | -0.012 | -0.005 |  | **0.004** | 0.090 | 0.077 |
| Cf_Stechlin_2011 | -0.005 | 0.006 | 0.010 | **0.027** |  | 0.107 | 0.071 |
| Cf_Stechlin_2012 | 0.010 | 0.000 | -0.018 | 0.009 | 0.013 |  | 0.244 |
| Cf_Stechlin_2018 | **0.025** | 0.004 | 0.006 | 0.019 | 0.019 | 0.012 |  |

**Table S3:** Matrix of pairwise θ between sympatric populations of Lake Breiter Luzin (*Coregonus albula* and *C. lucinensis*), sampled in different years (below diagonal), and their P-values (above diagonal).

|  | Ca_BLuzin_2011 | Ca_BLuzin_2012 | Cl_BLuzin_2011 |
| --- | --- | --- | --- |
| Ca_BLuzin_2011 |  | **0.032** | 0.433 |
| Ca_BLuzin_2012 | **0.024** |  | 0.077 |
| Cl_BLuzin_2011 | -0.003 | 0.013 |  |

**Table S4:** P-values of deviations from Hardy-Weinberg equilibrium per locus and population, with P<0.05 considered a significant deviation. The original alpha=0.05 was controlled by the false discovery rate for multiple tests.

| Locus/ Population | BWF1 | | BWF2 | | Cisco126 | | Cisco157 | | Cisco90 | | Cocl23 | | Sfo23 | | Sfo8 | | Str73 | | numbers of deviations | |  |
| --- | --- | --- | --- | --- | --- | --- | --- | --- | --- | --- | --- | --- | --- | --- | --- | --- | --- | --- | --- | --- | --- |
| Calbula_Stechlin | | 0.005 | | 0.000 | | 1.000 | | 1.000 | | 1.000 | | 0.000 | | 0.031 | | 0.000 | | 1.000 | | 5 | |
| Cfontanae_Stechlin | | 1.000 | | 0.020 | | 1.000 | | 1.000 | | 1.000 | | 0.040 | | 1.000 | | 0.000 | | 0.000 | | 4 | |
| Calbula_BLuzin | | 0.032 | | 1.000 | | 1.000 | | 1.000 | | 1.000 | | 1.000 | | 1.000 | | 1.000 | | 1.000 | | 1 | |
| Clucinensis_BLuzin | | 1.000 | | 0.001 | | 1.000 | | 1.000 | | 1.000 | | 1.000 | | 1.000 | | 1.000 | | 1.000 | | 1 | |
| Calbula_AS_Fegen | | 1.000 | | 1.000 | | 1.000 | | 1.000 | | 1.000 | | 1.000 | | 1.000 | | 1.000 | | 1.000 | | 0 | |
| Calbula_SS_Fegen | | 1.000 | | 1.000 | | 1.000 | | 1.000 | | 1.000 | | 1.000 | | 1.000 | | 1.000 | | 1.000 | | 0 | |
| Calbula_Asnen | | 1.000 | | 1.000 | | 1.000 | | 1.000 | | 1.000 | | 0.000 | | 1.000 | | 1.000 | | 1.000 | | 1 | |
| Calbula_Halsjön | | 1.000 | | 1.000 | | 1.000 | | 1.000 | | 1.000 | | 1.000 | | 1.000 | | 1.000 | | 1.000 | | 0 | |
| Calbula_Kalix | | 0.040 | | 1.000 | | 1.000 | | 1.000 | | 1.000 | | 1.000 | | 1.000 | | 1.000 | | 1.000 | | 1 | |
| Calbula_Vättern | | 1.000 | | 1.000 | | 1.000 | | 1.000 | | 1.000 | | 0.031 | | 1.000 | | 1.000 | | 1.000 | | 1 | |
| Calbula_Insko | | 0.005 | | 0.004 | | 0.000 | | 1.000 | | 1.000 | | 1.000 | | 0.019 | | 1.000 | | 0.000 | | 5 | |
| Calbula_Miedwie | | 0.040 | | 1.000 | | 0.031 | | 1.000 | | 1.000 | | 0.017 | | 1.000 | | 1.000 | | 0.031 | | 4 | |
| Calbula_Siecino | | 0.006 | | 1.000 | | 1.000 | | 1.000 | | 1.000 | | 1.000 | | 1.000 | | 1.000 | | 1.000 | | 1 | |
| Calbula_Oulujärvi | | 1.000 | | 1.000 | | 1.000 | | 1.000 | | 1.000 | | 1.000 | | 1.000 | | 1.000 | | 1.000 | | 0 | |
| Calbula_Jerisjärvi | | 1.000 | | 1.000 | | 1.000 | | 1.000 | | 1.000 | | 1.000 | | 1.000 | | 1.000 | | 1.000 | | 0 | |
| Calbula_Puruvesi | | 0.001 | | 1.000 | | 1.000 | | 1.000 | | 1.000 | | 1.000 | | 0.024 | | 0.036 | | 0.002 | | 4 | |
| Csardinella_YeniseiRiver | | 1.000 | | 1.000 | | 1.000 | | 1.000 | | 1.000 | | 1.000 | | 1.000 | | 1.000 | | 1.000 | | 0 | |
| Cmaraena_Bolmen | | 1.000 | | 1.000 | | 1.000 | | 1.000 | | 1.000 | | 1.000 | | 1.000 | | 1.000 | | 1.000 | | 0 | |
| Numbers of deviations | 7 | | 4 | | 2 | | 0 | | 0 | | 5 | | 3 | | 3 | | 4 | | 28 | |  |

**Table S5: Matrix of pairwise θ between 18 sympatric and allopatric *Coregonus* populations**

Matrix of pairwise θ between 18 sympatric and allopatric *Coregonus* populations from 15 lakes or rivers in Germany, Sweden, Poland, Finland and Russia (below diagonal), and their P-values as obtained by G-tests (above diagonal). Strong structure between populations is indicated in bold. AS=autumn-spawner, SS=spring-spawner

|  | Calbula  Stechlin | Cfontanae  Stechlin | Calbula  BLuzin | Clucinensis  BLuzin | Calbula_AS  Fegen | Calbula_SS  Fegen | Calbula  Åsnen | Calbula  Hålsjön | Calbula  Kalix | Calbula  Vättern | Calbula  Ińsko | Calbula  Miedwie | Calbula  Siecino | Calbula  Oulujärvi | Calbula  Jerisjärvi | Calbula  Puruvesi | Csardinella  YeniseiRiver | Cmaraena  Bolmen |
| --- | --- | --- | --- | --- | --- | --- | --- | --- | --- | --- | --- | --- | --- | --- | --- | --- | --- | --- |
| Calbula Stechlin |  | **0.0006** | **<0.0001** | **<0.0001** | **<0.0001** | **<0.0001** | **<0.0001** | **<0.0001** | **<0.0001** | **<0.0001** | **<0.0001** | **<0.0001** | **<0.0001** | **<0.0001** | **<0.0001** | **<0.0001** | **<0.0001** | **<0.0001** |
| Cfontanae  Stechlin | **0.0054** |  | **<0.0001** | **<0.0001** | **<0.0001** | **<0.0001** | **<0.0001** | **<0.0001** | **<0.0001** | **<0.0001** | **<0.0001** | **<0.0001** | **<0.0001** | **<0.0001** | **<0.0001** | **<0.0001** | **<0.0001** | **<0.0001** |
| Calbula  BLuzin | **0.05** | **0.06** |  | 0.63 | **<0.0001** | **<0.0001** | **<0.0001** | **<0.0001** | **<0.0001** | **<0.0001** | **<0.0001** | **<0.0001** | **<0.0001** | **<0.0001** | **<0.0001** | **<0.0001** | **<0.0001** | **<0.0001** |
| Clucinensis  BLuzin | **0.04** | **0.04** | -0.0012 |  | **<0.0001** | **<0.0001** | **<0.0001** | **<0.0001** | **<0.0001** | **<0.0001** | **<0.0001** | **<0.0001** | **<0.0001** | **<0.0001** | **<0.0001** | **<0.0001** | **<0.0001** | **<0.0001** |
| Calbula_AS  Fegen | **0.18** | **0.17** | **0.20** | **0.19** |  | **<0.0001** | **<0.0001** | **<0.0001** | **<0.0001** | **<0.0001** | **<0.0001** | **<0.0001** | **<0.0001** | **<0.0001** | **<0.0001** | **<0.0001** | **<0.0001** | **<0.0001** |
| Calbula_SS  Fegen | **0.19** | **0.19** | **0.21** | **0.19** | **0.06** |  | **<0.0001** | **<0.0001** | **<0.0001** | **<0.0001** | **<0.0001** | **<0.0001** | **<0.0001** | **<0.0001** | **<0.0001** | **<0.0001** | **<0.0001** | **<0.0001** |
| Calbula  Åsnen | **0.12** | **0.12** | **0.16** | **0.15** | **0.15** | **0.17** |  | **<0.0001** | **<0.0001** | **<0.0001** | **<0.0001** | **<0.0001** | **<0.0001** | **<0.0001** | **<0.0001** | **<0.0001** | **<0.0001** | **<0.0001** |
| Calbula  Hålsjön | **0.20** | **0.19** | **0.24** | **0.22** | **0.14** | **0.15** | **0.13** |  | **<0.0001** | **<0.0001** | **<0.0001** | **<0.0001** | **<0.0001** | **<0.0001** | **<0.0001** | **<0.0001** | **<0.0001** | **<0.0001** |
| Calbula  Kalix | **0.06** | **0.06** | **0.08** | **0.07** | **0.17** | **0.18** | **0.09** | **0.18** |  | **0.0001** | **<0.0001** | **<0.0001** | **<0.0001** | **<0.0001** | **<0.0001** | **<0.0001** | **<0.0001** | **<0.0001** |
| Calbula  Vättern | **0.06** | **0.07** | **0.07** | **0.06** | **0.17** | **0.19** | **0.10** | **0.18** | **0.0135** |  | **<0.0001** | **<0.0001** | **<0.0001** | **<0.0001** | **<0.0001** | **<0.0001** | **<0.0001** | **<0.0001** |
| Calbula  Ińsko | **0.05** | **0.07** | **0.05** | **0.04** | **0.20** | **0.20** | **0.15** | **0.25** | **0.06** | **0.07** |  | **<0.0001** | **0.0001** | **<0.0001** | **<0.0001** | **<0.0001** | **<0.0001** | **<0.0001** |
| Calbula  Miedwie | **0.04** | **0.07** | **0.06** | **0.06** | **0.21** | **0.22** | **0.12** | **0.24** | **0.05** | **0.05** | **0.02** |  | **0.0060** | **<0.0001** | **<0.0001** | **<0.0001** | **<0.0001** | **<0.0001** |
| Calbula  Siecino | **0.05** | **0.08** | **0.08** | **0.07** | **0.21** | **0.21** | **0.10** | **0.24** | **0.05** | **0.06** | **0.03** | **0.0117** |  | **<0.0001** | **<0.0001** | **<0.0001** | **<0.0001** | **<0.0001** |
| Calbula  Oulujärvi | **0.05** | **0.05** | **0.06** | **0.05** | **0.16** | **0.17** | **0.07** | **0.16** | **0.03** | **0.04** | **0.05** | **0.05** | **0.05** |  | **<0.0001** | **<0.0001** | **<0.0001** | **<0.0001** |
| Calbula  Jerisjärvi | **0.11** | **0.12** | **0.12** | **0.12** | **0.28** | **0.28** | **0.18** | **0.30** | **0.12** | **0.11** | **0.12** | **0.11** | **0.12** | **0.08** |  | **<0.0001** | **<0.0001** | **<0.0001** |
| Calbula  Puruvesi | **0.13** | **0.13** | **0.12** | **0.14** | **0.26** | **0.27** | **0.17** | **0.26** | **0.10** | **0.10** | **0.12** | **0.10** | **0.13** | **0.10** | **0.15** |  | **<0.0001** | **<0.0001** |
| Csardinella  YeniseiRiver | **0.10** | **0.10** | **0.12** | **0.12** | **0.26** | **0.27** | **0.19** | **0.28** | **0.11** | **0.10** | **0.14** | **0.10** | **0.14** | **0.11** | **0.13** | **0.10** |  | **<0.0001** |
| Cmaraena  Bolmen | **0.23** | **0.24** | **0.22** | **0.23** | **0.37** | **0.37** | **0.33** | **0.40** | **0.25** | **0.23** | **0.24** | **0.22** | **0.26** | **0.24** | **0.26** | **0.21** | **0.19** |  |

**Table S6**: Overview of systematic differences in repeat length of nine bi-allelic microsatellite markers for eight cisco individuals from six Swedish populations, analysed either on an ABI3130xl sequencer (Applied Biosystems) (SWE) or an Beckmann Coulter CEQ 8000 sequencer (GER). The differences in read length were added to the original data for the Swedish populations included in the study.

| Marker | BWF1 |  |  |  | BWF2 |  |  |  | Cisco126 | |  |  | Cisco157 | |  |  | Cisco90 |  |  |  |
| --- | --- | --- | --- | --- | --- | --- | --- | --- | --- | --- | --- | --- | --- | --- | --- | --- | --- | --- | --- | --- |
| Population | SWE | GER | SWE | GER | SWE | GER | SWE | GER | SWE | GER | SWE | GER | SWE | GER | SWE | GER | SWE | GER | SWE | GER |
| L.Fegen_AS | 221 | 226 | 288 | 293 | 165 | 168 | 165 | 168 | 208 | 212 | 208 | 212 | 116 | 120 | 126 | 130 | 129 | 135 | 129 | 135 |
| L.Fegen_SS | 221 | 226 | 298 | 303 | 165 | 168 | 229 | 231 | 208 | 212 | 208 | 212 | 116 | 120 | 116 | 120 | 129 | 135 | 133 | 139 |
| L.Mälaren | 208 | 212 | 222 | 227 | 165 | 168 | 205 | 208 | 200 | 204 | 208 | 212 | 116 | 120 | 138 | 142 | 115 | 121 | 119 | 125 |
| L.Stora Hälsjön | 221 | 226 | 232 | 237 | 165 | 168 | 233 | 235 | 202 | 206 | 210 | 214 | 116 | 120 | 116 | 120 | 121 | 127 | 129 | 135 |
| L.Stora Hälsjön | 228 | 233 | 232 | 237 | 165 | 168 | 233 | 235 | 208 | 212 | 210 | 214 | 116 | 120 | 126 | 130 | 121 | 127 | 125 | 131 |
| L.Asnen | 221 | 226 | 290 | 295 | 165 | 168 | 245 | 248 | 202 | 206 | 208 | 212 | 116 | 120 | 116 | 120 | 119 | 125 | 123 | 129 |
| L.Siljan | 212 | 217 | 216 | 221 | 143 | 146 | 177 | 180 | 202 | 206 | 208 | 212 | 116 | 120 | 152 | 158 | 111 | 117 | 123 | 129 |
| L.Siljan | 208 | 213 | 220 | 225 | 203 | 206 | 203 | 206 | 202 | 206 | 204 | 208 | 116 | 120 | 156 | 162 | 119 | 125 | 119 | 125 |
|  |  |  |  |  |  |  |  |  |  |  |  |  |  |  |  |  |  |  |  |  |
| Difference |  | 5 |  | 5 |  | 3 |  | 3 |  | 4 |  | 4 |  | 4 |  | 4 |  | 6 |  | 6 |
|  |  |  |  |  |  |  |  |  |  |  |  |  |  |  |  |  |  |  |  |  |
|  |  |  |  |  |  |  |  |  |  |  |  |  |  |  |  |  |  |  |  |  |
| Marker | Cocl23 |  |  |  | Sfo23 |  |  |  | Sfo8 |  |  |  | Str73 |  |  |  |  |  |  |  |
| Population | SWE | GER | SWE | GER | SWE | GER | SWE | GER | SWE | GER | SWE | GER | SWE | GER | SWE | GER |  |  |  |  |
| L.Fegen_AS | 242 | 248 | 244 | 250 | 157 | 159 | 229 | 231 | 192 | 196 | 192 | 196 | 130 | 134 | 130 | 134 |  |  |  |  |
| L.Fegen_SS | 242 | 248 | 242 | 248 | 169 | 171 | 205 | 207 | 192 | 196 | 200 | 204 | 130 | 134 | 130 | 134 |  |  |  |  |
| L.Mälaren | 242 | 248 | 244 | 250 | 177 | 179 | 177 | 179 | 192 | 196 | 200 | 204 | 130 | 134 | 130 | 134 |  |  |  |  |
| L.Stora Hälsjön | 242 | 248 | 242 | 248 | 157 | 159 | 195 | 197 | 200 | 204 | 200 | 204 | 130 | 134 | 130 | 134 |  |  |  |  |
| L.Stora Hälsjön | 242 | 248 | 242 | 248 | 193 | 195 | 213 | 215 | 192 | 196 | 206 | 210 | 130 | 134 | 130 | 134 |  |  |  |  |
| L.Asnen | 242 | 248 | 242 | 248 | 171 | 173 | 183 | 185 | 192 | 196 | 200 | 204 | 130 | 134 | 130 | 134 |  |  |  |  |
| L.Siljan | 230 | 236 | 242 | 248 | 179 | 181 | 217 | 219 | 196 | 200 | 200 | 204 | 130 | 134 | 132 | 136 |  |  |  |  |
| L.Siljan | 230 | 236 | 230 | 236 | 163 | 165 | 165 | 167 | 192 | 196 | 196 | 200 | 130 | 134 | 130 | 134 |  |  |  |  |
|  |  |  |  |  |  |  |  |  |  |  |  |  |  |  |  |  |  |  |  |  |
| SDifference |  | 6 |  | 6 |  | 2 |  | 2 |  | 4 |  | 4 |  | 4 |  | 4 |  |  |  |  |

**Figure S1**: Overview on number of alleles per nine microsatellite loci, for 655 individuals of Baltic ciscoes from 18 lake and river populations in Germany, Sweden, Poland, Finland and Russia.


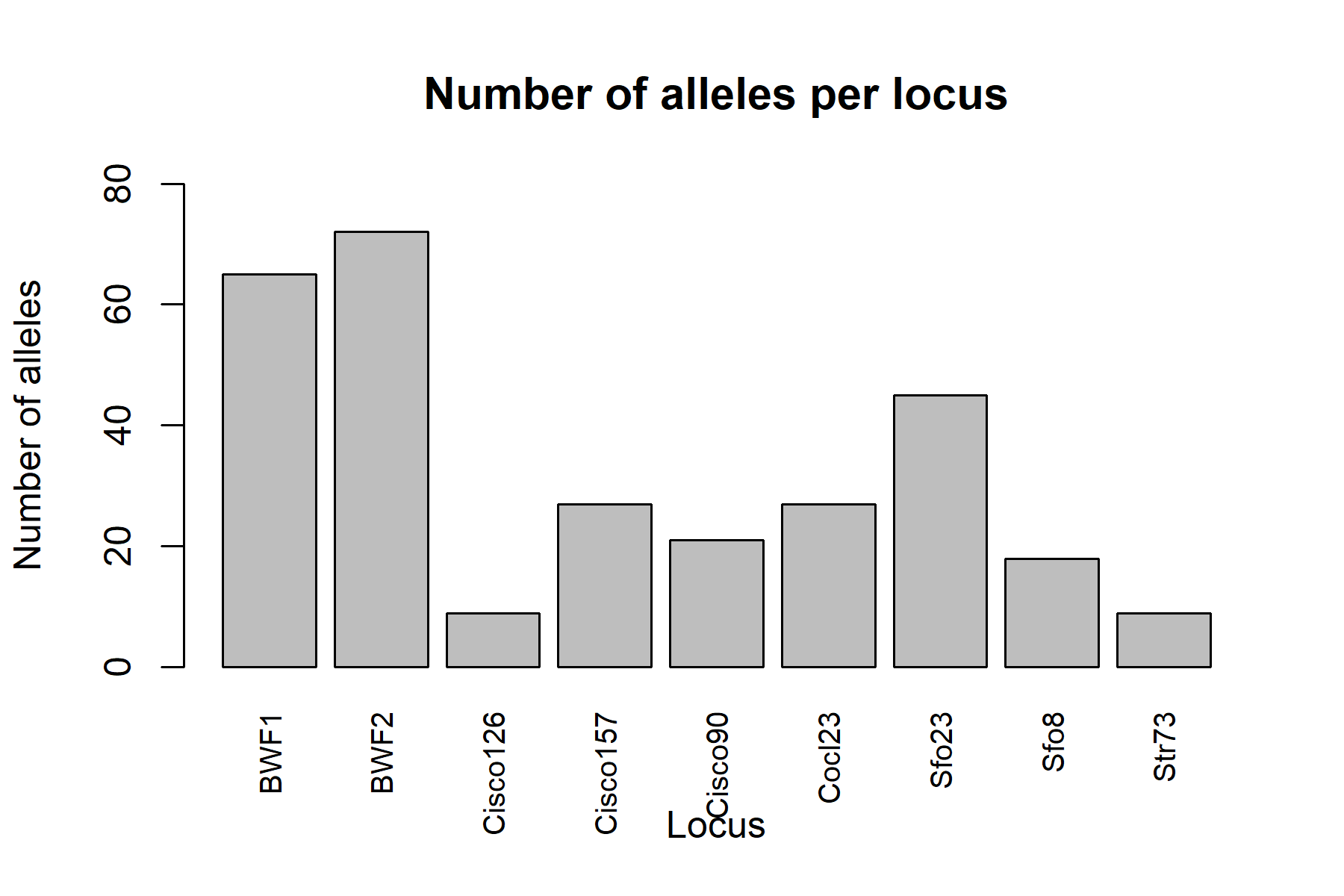


**Figure S2**: Separate biplots of 18 *Coregonus* populations in reduced space along axes 1 and 2 of the Principal Coordinate Analysis. The axes dimensions are identically scaled to facilitate comparison of location and extent of population diversification.


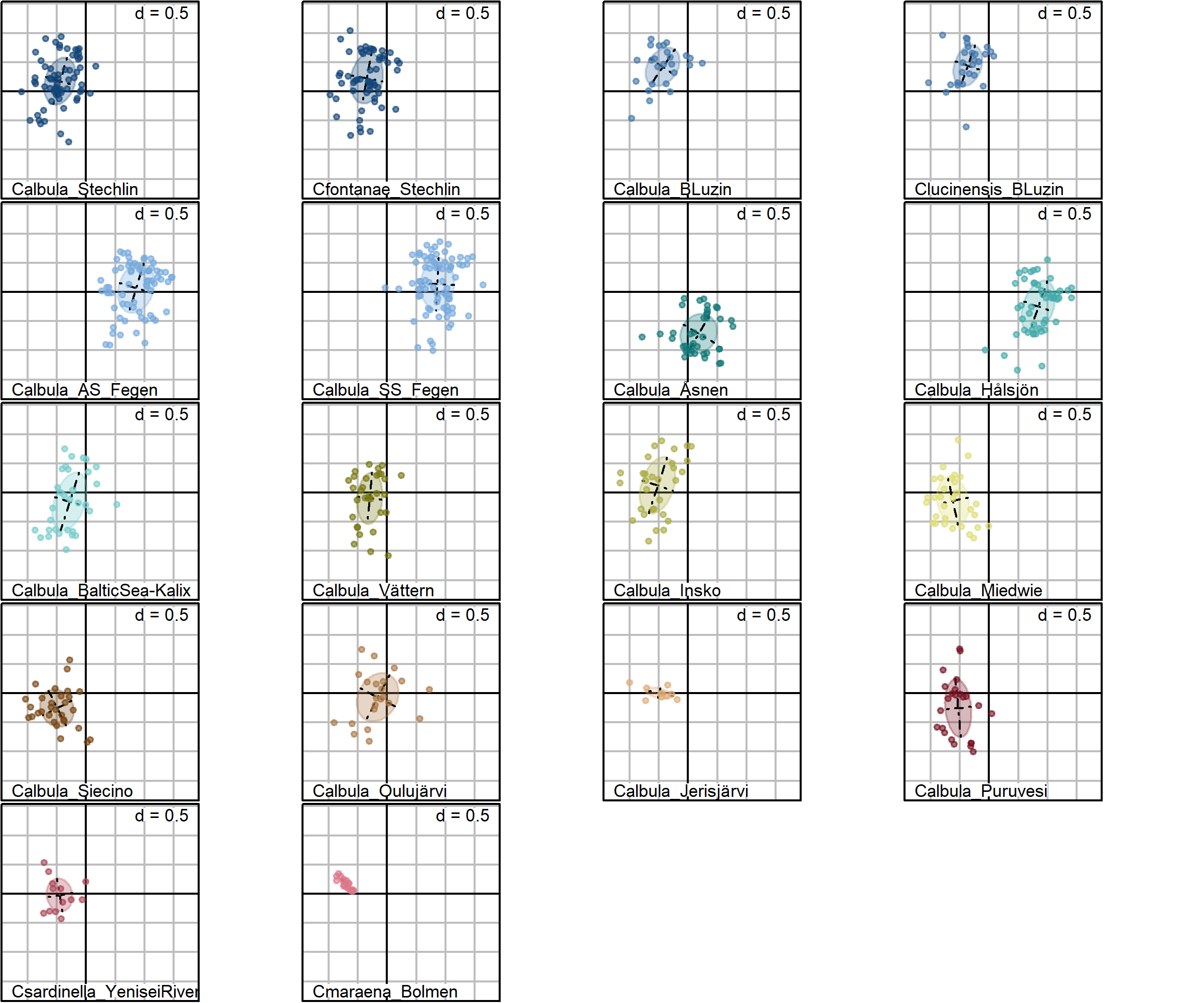


**Figure S3**: Plot of cross-entropy vs. a range of 1 to 15 ancestral populations, as estimated by the R-package LEA.


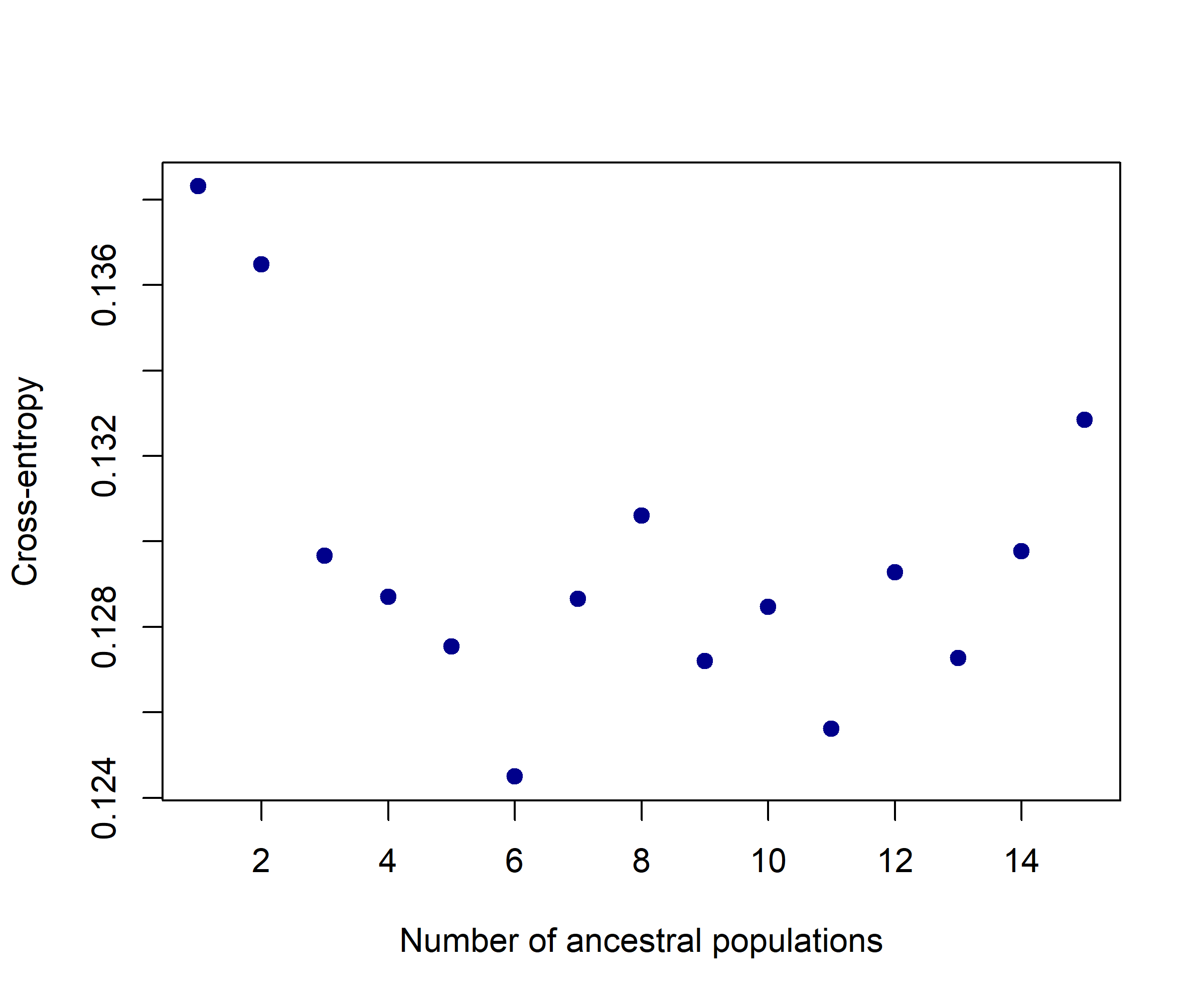

Supplement: Supplementary file 1 — Additional file 1: Table S1. Proportion of missing genotypes among the nine microsatellites (columns) in the 18 Coregonus populations (rows). Individuals with more than a single missing locus were omitted from subsequent statistical analyses. AS = autumn-spawner, SS = spring-Spawner. Table S2. Matrix of pairwise θ between sympatric populations of Lake Stechlin (Coregonus albula and C. fontanae), sampled in different years (below diagonal), and their lower 95% confidence intervals (above diagonal). Weak structure between (sub)populations is indicated by the lower CI including zero. Table S3. Matrix of pairwise θ between sympatric populations of Lake Breiter Luzin (Coregonus albula and C. lucinensis), sampled in different years (below diagonal), and their lower 95% confidence intervals (above diagonal). Weak structure between (sub)populations is indicated by the lower CI including zero. Table S4. P-values of deviations from Hardy–Weinberg equilibrium per locus and population, with P < 0.05 considered a significant deviation. The original alpha = 0.05 was controlled by the false discovery rate for multiple tests. Table S5. Matrix of pairwise θ between 18 sympatric and allopatric Coregonus populations from 15 lakes or rivers in Germany, Sweden, Poland, Finland and Russia (below diagonal), and their P-values as obtained by G-tests (above diagonal). Strong structure between populations is indicated in bold. AS = autumn-spawner, SS = spring-spawner. Table S6. Overview of systematic differences in repeat length of nine bi-allelic microsatellite markers for eight cisco individuals from six Swedish populations, analysed either on an ABI3130xl sequencer (Applied Biosystems) (SWE) or an Beckmann Coulter CEQ 8000 sequencer (GER). The differences in read length were added to the original data for the Swedish populations included in the study. Figure S1. Overview on number of alleles per nine microsatellite loci, for 655 individuals of Baltic ciscoes from 18 lake and river populat [file 12862_2021_1920_MOESM1_ESM.docx]
